# Platelet-instructed SPP1^+^ macrophages drive myofibroblast activation in fibrosis in a CXCL4-dependent manner

**DOI:** 10.1016/j.celrep.2023.112131

**Published:** 2023-02-18

**Authors:** Konrad Hoeft, Gideon J.L. Schaefer, Hyojin Kim, David Schumacher, Tore Bleckwehl, Qingqing Long, Barbara Mara Klinkhammer, Fabian Peisker, Lars Koch, James Nagai, Maurice Halder, Susanne Ziegler, Elisa Liehn, Christoph Kuppe, Jennifer Kranz, Sylvia Menzel, Ivan Costa, Adam Wahida, Peter Boor, Rebekka K. Schneider, Sikander Hayat, Rafael Kramann

**Affiliations:** 1 Division of Nephrology and Clinical Immunology, RWTH Aachen University, Aachen, Germany; 2 Institute of Experimental Medicine and Systems Biology, RWTH Aachen University, Aachen, Germany; 3 Department of Anesthesiology, RWTH Aachen University, Aachen, Germany; 4 Department of Pathology, RWTH Aachen University, Aachen, Germany; 5 Institute for Computational Genomics, RWTH Aachen University Hospital, Aachen, Germany; 6 Joint Research Center for Computational Biomedicine, RWTH Aachen University Hospital, Aachen, Germany; 7 Institute for Molecular Medicine, University of South Denmark, Odense, Denmark; 8 Department of Urology, RWTH Aachen University, Aachen, Germany; 9 Department of Urology and Kidney Transplantation, Martin-Luther-University, Halle (Saale), Germany; 10 Institute of Metabolism and Cell Death, Helmholtz Zentrum München, Neuherberg, Germany; 11 Division of Gynecological Oncology, National Center for Tumor Diseases (NCT), Heidelberg, Germany; 12 Department of Hematology, Erasmus MC Cancer Institute, Rotterdam, the Netherlands; 13 Department of Cell Biology, Institute for Biomedical Technologies, RWTH Aachen University, Aachen, Germany; 14 Department of Internal Medicine, Nephrology and Transplantation, Erasmus Medical Center, Rotterdam, the Netherlands

**Keywords:** fibrosis, innate immunity, heart failure, chronic kidney disease, platelets, SPP1 macrophages, CXCL4, PF4, myocardial infarction, SPP1

## Abstract

Fibrosis represents the common end stage of chronic organ injury independent of the initial insult, destroying tissue architecture and driving organ failure. Here we discover a population of profibrotic macrophages marked by expression of *Spp1*, *Fn1*, and *Arg1* (termed *Spp1* macrophages), which expands after organ injury. Using an unbiased approach, we identify the chemokine (C-X-C motif) ligand 4 (CXCL4) to be among the top upregulated genes during profibrotic *Spp1* macrophage differentiation. *In vitro* and *in vivo* studies show that loss of *Cxcl4* abrogates profibrotic *Spp1* macrophage differentiation and ameliorates fibrosis after both heart and kidney injury. Moreover, we find that platelets, the most abundant source of CXCL4 *in vivo*, drive profibrotic *Spp1* macrophage differentiation. Single nuclear RNA sequencing with ligand-receptor interaction analysis reveals that macrophages orchestrate fibroblast activation via *Spp1*, *Fn1*, and *Sema3* crosstalk. Finally, we confirm that *Spp1* macrophages expand in both human chronic kidney disease and heart failure.

## Highlights

• ECM regulator scoring identifies a *SPP1*^+^ macrophage subset in myocardial infarction
• Chemokine CXCL4 drives *SPP1*^+^ macrophage activation and macrophage-fibroblast crosstalk
• Platelet-derived CXCL4 mediates *SPP1*^+^ macrophage activation and organ fibrosis
• *SPP1*^*+*^ macrophages expand in human heart failure and chronic kidney disease

## Graphical abstract

**Figure:**
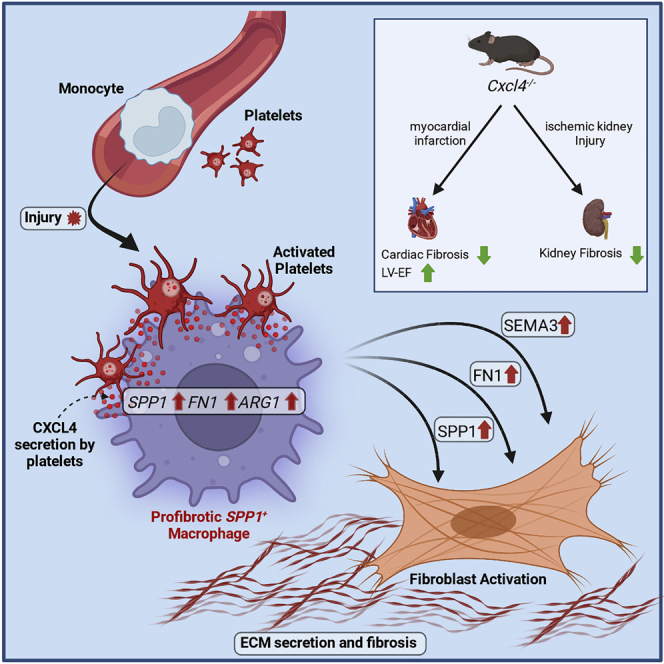

## Teaser

By ranking immune cells after myocardial infarction based on ECM regulator expression, Hoeft et al. find an *SPP1*^+^ macrophage subset. They identify the chemokine CXCL4 as critical for *SPP1*^+^ macrophage activation, macrophage-fibroblast crosstalk, and organ fibrosis. They confirm expansion of *SPP1*^+^ macrophages in human heart failure and chronic kidney disease.

## Introduction

Fibrosis represents the common response of organs and tissues to virtually all chronic repetitive injuries.^1^ Although the initial fibrotic response is crucial for tissue repair and preservation of organ integrity, continued deposition of extracellular matrix (ECM) can lead to maladaptive remodeling and deterioration of organ function. As such, fibrosis is considered to be accountable for up to 45% of all deaths in the industrialized world.^2^ Synthesizing the major fraction of ECM, myofibroblasts are regarded as the primary drivers of fibrotic disease and organ dysfunction. Recent studies show that the large majority of myofibroblasts originate from resident mesenchymal cells such as fibroblasts and pericytes.^3^^,^^4^^,^^5^^,^^6^ Despite these new insights, we still lack a precise understanding of both the molecular and cellular cues that initiate mesenchymal cell activation and myofibroblast differentiation.

Immune cells are one of the key players shaping the development of fibrosis, which can either ameliorate or aggravate tissue remodeling.^7^^,^^8^ Intriguingly, despite their central role in fibrogenesis, immune cells neither secrete high amounts of ECM nor represent a cellular source of the fibrosis-defining myofibroblasts.^6^^,^^9^^,^^10^^,^^11^^,^^12^ Instead, immune cells are thought to control fibrosis via regulation of mesenchymal cell activation and ECM degradation. In particular, mononuclear phagocytes (MPCs: monocytes, macrophages and dendritic cells) display high plasticity after injury and in fibrosis with strong dynamic changes in both population size and composition.^13^^,^^14^ Immediately after injury, massive infiltration of M1-like Ly6c2^hi^ monocytes defines an early inflammatory phase (day 0–2), followed by expansion of M2-like macrophages during the remodeling phase (day 3–7), which coincides with the emergence of fibrosis. In line with this concept, several studies have highlighted that MPC can exert opposing effects depending on the subpopulation, time, and type of injury, either driving fibrosis and organ failure or aiding in tissue repair.^15^^,^^16^^,^^17^^,^^18^ While the advent of RNA and single-cell RNA sequencing (scRNA-seq) has helped elucidate MPC heterogeneity beyond traditional M1/M2 paradigms,^18^^,^^19^^,^^20^^,^^21^^,^^22^ the signals driving profibrotic immune cell differentiation and subsequent fibroblast crosstalk remain ill-defined.

In this study, we leveraged scRNA-seq data from a murine myocardial infarction time series to identify a profibrotic macrophage population defined by expression of *Spp1* and elucidate the molecular cues that drive profibrotic macrophage differentiation. We confirm that expansion of *Spp1*^*+*^ macrophages after injury is dependent on the chemokine (C-X-C motif) ligand 4 (*Cxcl4*) and dissect its role using genetic perturbation, bone marrow transplantation, and single nuclear RNA sequencing (snRNA-seq). Our results indicate that *Spp1*^*+*^ macrophages expand in response to both platelet- and monocyte-secreted CXCL4 and subsequently orchestrate tissue remodeling via fibroblast crosstalk.

## Results

### ECM regulator scoring identifies a macrophage subpopulation with profibrotic properties

**Figure 1:**
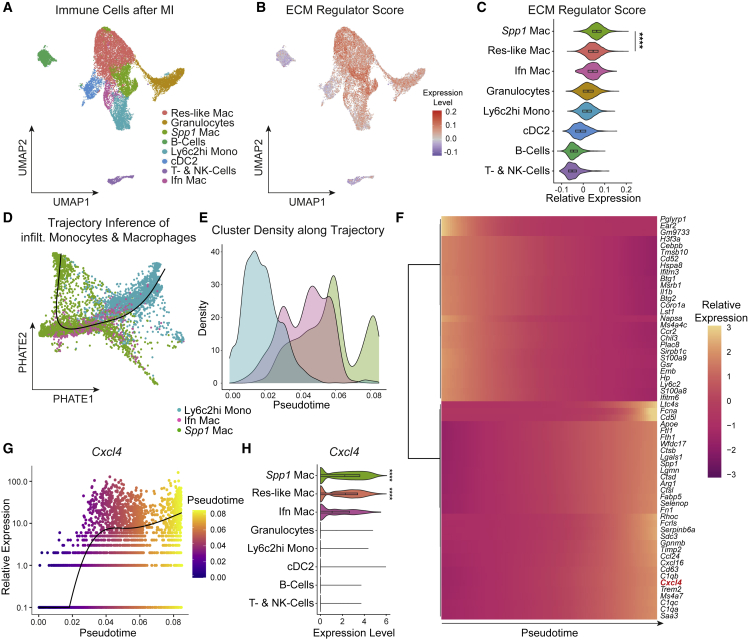
ECM regulator scoring identifies profibrotic *Spp1*^*+*^ macrophages (A) UMAP embedding of 17,690 Cd45^+^ immune cells from murine heart tissue at different timepoints after myocardial infarction from Forte et al.^23^ Labels refer to clusters. Res-like Mac: resident-like macrophages, *Spp1* Mac: *Spp1*^+^ macrophages, Ly6c2hi Mono: Ly6c2 high monocytes, cDC2: conventional dendritic cells type 2, T- & NK-cells: T-cells and natural killer cells, Ifn Mac: Interferon-induced macrophages. (B) Featureplot of ECM regulator score on the UMAP embedding shown in (A). (C) ECM regulator score stratified by immune cell type. (D) Fitted Slingshot pseudotime trajectory for infiltrating MPC on a PHATE dimensionality reduction. (E) Line graph showing Cluster Density (in % of all cluster cells) of infiltrating MPC along pseudotime. (F) Heatmap of top dynamically expressed genes along pseudotime. (G) Expression of *Cxcl4* along pseudotime. Each dot represents an individual pseudotime-ordered cell. (H) *Cxcl4* expression stratified by immune cell type. For (C), a two-tailed unpaired t test (*Spp1* Mac versus Res-like Mac) was computed. For (H) p values from MAST (Seurat, FindAllMarkers) are displayed. ^∗∗∗∗^p < 0.0001. See also Figures S1 and S2.

To characterize and disentangle profibrotic immune cell populations, we sub-clustered leukocytes from a publicly available murine scRNA-seq time course of left ventricular myocardial infarction (MI) (Figures S1A and S1B, Table S1).^23^ Clustering and annotation revealed all major immune cell populations in MI (Figures 1A and S1C, Table S1). While the early, inflammatory phase (day 1–3) after MI was characterized by expansion of Ly6c2^hi^ monocytes and granulocytes, the later remodeling phase (day 3–7) was marked by the expansion of resident-like macrophages, as well as a second macrophage cluster with high expression of *Spp1*, *Arg1*, and *Fn1* (hereafter *Spp1*^*+*^ macrophages) (Figures S1C and S1D). To identify profibrotic immune cell populations in an unbiased manner, we scored cells according to their expression of a profibrotic ECM regulator gene set as defined by the matrisome project (Figure 1B).^24^ By assigning these signatures, we found that ECM regulator expression was highest in *Spp1*^*+*^ macrophages, which were additionally defined by the expression of profibrotic genes (*Spp1*, *Fn1*) (Figure 1C). As expected, while MPC displayed the highest ECM regulator scores, they did not express core ECM components (*core matrisome*: collagens, glycoproteins, and proteoglycans) at a considerable level (Figures S1E and S1F), suggesting that their role in fibrosis is rather regulatory.^25^

Next, to unravel the signaling pathways that characterize *Spp1*^*+*^ macrophage activation, we imputed pathway activity of immune cells using PROGENy.^26^ As expected, granulocytes, Ly6c2hi monocytes, and Ifn Mac displayed strong activity of proinflammatory tumor necrosis factor (TNF), Janus kinase-signal transducer and activator of transcription protein (JAK-STAT), and nuclear factor (NF)-κB pathways, while Res-like Mac displayed low proinflammatory pathway activity, but instead increased WNT and tumor necrosis factor-related apoptosis-inducing ligand (TRAIL) signaling (Figure S2A). These findings are in line with the current concept, that the initial inflammatory response is driven by infiltrating macrophages, monocytes, and granulocytes, while resident macrophages control the resolution of inflammation and subsequent tissue remodeling.^27^^,^^28^ In contrast, *Spp1*^+^ macrophages displayed strong hypoxia and increased transforming growth factor (TGF)β, NF-κB, and TNF signaling (Figure S2A), emphasizing a profibrotic, latent proinflammatory activation of *Spp1*^+^ macrophages. To further identify the transcription factors controlling profibrotic *Spp1*^+^ macrophage gene expression, we inferred DoRothEA transcription factor (TF) activity.^29^^,^^30^ In line with our pathway analysis, *Spp1*^+^ macrophages were characterized by high Hif1a, Myc, and Spi1 TF activity (Figure S2B), central transcription factors in hypoxia and fibrosis.^31^^,^^32^^,^^33^ Of note, both Hif1a and Myc have previously been proposed as bona fide M1 and M2 markers, highlighting that the observed *Spp1*^+^ macrophage signature is not sufficiently captured by the traditional M1/M2 paradigm.^31^^,^^34^ As recent studies have emphasized the role of *TREM2*^*+*^ macrophages in wound healing, we examined whether *Spp1*^*+*^ macrophages correspond to *TREM2*^*+*^ macrophages.^35^ While *Spp1*^*+*^ macrophages did express *Trem2*, this expression pattern was not specific, with higher *Trem2* expression in resident-like macrophages (Figure S2C).

### *Cxcl4* correlates with ECM regulator scores across immune cells

To further characterize *Spp1*^*+*^ macrophages as well as genes that drive their differentiation, we performed pseudotime trajectory inference analysis using PHATE dimensionality reduction and Slingshot.^36^ Based on *Spp1*^*+*^ macrophage kinetics after MI (Figure S1D) and recent literature,^37^ we hypothesized that *Spp1*^*+*^ macrophages are monocyte-derived and therefore subsetted infiltrating MPC (Ly6c2hi monocytes, Ifn macrophages, *Spp1*^*+*^ macrophages) for subsequent analysis. Using these three clusters, PHATE dimensionality reduction and Slingshot analysis identified one trajectory for Ly6c2hi monocyte to *Spp1*^*+*^ macrophage differentiation via Ifn Mac as an intermediate cluster (Figures 1D, 1E, and S2D). Imputing differentially expressed genes along pseudotime revealed that *Spp1*^*+*^ macrophage differentiation was associated with downregulation of inflammatory genes (*Il1b*, *S100a8*, *Ifitm3*), and upregulation of profibrotic genes (*Spp1*, *Timp2*) (Figure 1F, Table S1). Interestingly, we identified the chemokine (C-X-C motif) ligand 4 (*Cxcl4*), also known as platelet factor 4, to rank among the top differentially expressed genes along pseudotime (Figures 1F and 1G). To verify *Cxcl4* as a potential driver of a profibrotic immune cell signature independent of our assumption on *Spp1*^*+*^ macrophage ontogeny for trajectory inference analysis, we correlated ECM regulator scores with gene expression across all immune cells. As expected, *Cxcl4* ranked again among the top genes (rank = 7) correlating with a profibrotic ECM regulator signature (Figure S2E). In line with these data, *Cxcl4* was exclusively co-expressed in macrophages with high ECM regulator signatures (Figure 1H), with highest expression in *Spp1*^*+*^ macrophages.

### *Spp1*^*+*^ macrophages map to an ECM remodeling trajectory of a framework dataset of monocyte-derived macrophage activation

To validate our findings of *Spp1*^*+*^ macrophages as potential drivers of tissue remodeling, we compared monocyte and macrophage populations with a recently published single-cell atlas of monocyte-derived macrophage activation states^38^ using Symphony.^39^ Consistent with previous work,^27^^,^^28^ Reference mapping indicated that resident-like macrophages map to a phagocytic trajectory (clusters Late P1 and Final P1) of macrophage activation, while infiltrating Ly6c2hi monocytes mapped to an inflammatory trajectory (cluster Final P3, Figures S2F and S2G). In contrast, *Spp1*^*+*^ macrophages mapped largely to an intermediate macrophage cluster within a (tissue) remodeling trajectory, validating our assignment of *Spp1*^*+*^ macrophages as potential drivers of tissue remodeling (Figures S2F and S2G). More importantly, Sanin et al. independently identified *Cxcl4* as one of the top upregulated genes along the (tissue) remodeling trajectory.^38^ These findings not only confirm *Spp1*^+^ macrophages as potential drivers of tissue remodeling but point toward an organ-independent function of *Cxcl4* driving profibrotic activation of macrophages.

### Loss of CXCL4 abrogates a profibrotic *Spp1*^*+*^ macrophage signature *in vitro*

**Figure 2:**
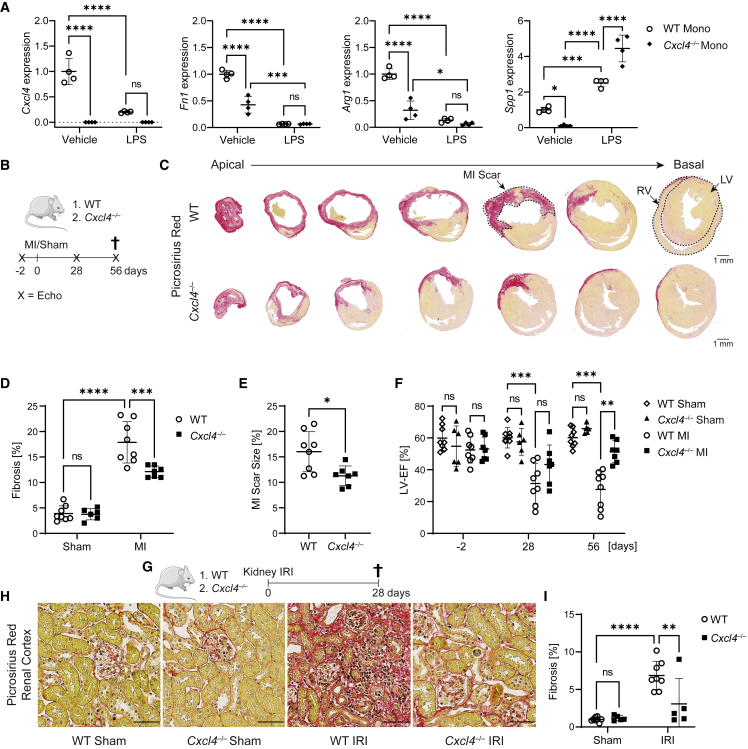
Genetic loss of *Cxcl4* mitigates organ fibrosis (A) RT-qPCR analysis for *Cxcl4*, *Fn1*, *Arg1,* and *Spp1* in CD11b^+^ monocytes isolated from WT or *Cxcl4*^*−/−*^ PBMCs after stimulation with vehicle or LPS (n = 4). Mono, monocytes. (B) Design of myocardial infarction experiments. (C) Picrosirius red stained serial heart sections over seven levels from WT and *Cxcl4*^*−/−*^ mice after MI. ECM is stained red. LV, left ventricle; RV, right ventricle. Scale bar = 1 mm. (D) Fibrosis of serial heart sections in WT sham, *Cxcl4*^*−/−*^ sham, WT MI, and *Cxcl4*^*−/−*^ MI mice based on quantification of serial heart sections shown in (C). Quantification by spectral thresholding analysis of red ECM (WT Sham = 8; *Cxcl4*^*−/−*^ Sham = 6; WT MI = 8; *Cxcl4*^*−/−*^ MI = 7). (E) MI scar sizes in WT and *Cxcl4*^*−/−*^ mice based on quantification of serial heart sections shown in (C). (F) Left ventricular ejection fraction (Simpsons) 2 days before, as well as 28 and 56 days after MI or sham surgery in WT and *Cxcl4*^*−/−*^ mice. (G) Experimental design of IRI experiments. (H) Representative images of picrosirius red stained cortical kidney sections from WT and *Cxcl4*^*−/−*^ mice after sham or IRI surgery. Scale bar = 50 μm. (I) Kidney cortex fibrosis (in % of cortex area) after sham or IRI surgery by quantification of red ECM of scans shown in (H) (WT mice = 8, *Cxcl4*^*−/−*^ mice = 5). All quantitative data are shown as mean ± SD. For (A), (D), (F), and (I), two-way ANOVA was computed using Tukey corrections. For (E), a two-tailed unpaired t test was performed. ^∗^p < 0.05, ^∗∗^p < 0.01, ^∗∗∗^p < 0.001, ^∗∗∗∗^p < 0.0001.

To verify whether *Cxcl4* is sufficient to induce the identified profibrotic *Spp1* signature in monocytes, we isolated CD11b^+^ monocytes from peripheral blood of wild-type (WT) and *Cxcl4*^*−/−*^ mice^40^ by magnetic cell isolation and incubated them in the absence or presence of LPS. Real-time quantitative polymerase chain reaction (RT-qPCR) analysis confirmed loss of *Cxcl4* in *Cxcl4*^*−/−*^ CD11b^+^ monocytes (Figure 2A). In accordance with our hypothesis, WT CD11b^+^ monocytes expressed significantly higher levels of the aforementioned profibrotic marker trio *Fn1*, *Arg1*, and *Spp1* (Figure S1C) in comparison to *Cxcl4*^*−/−*^ CD11b^+^ monocytes at baseline (Figure 2A). Proinflammatory polarization with LPS suppressed the expression of *Cxcl4*, *Arg1,* and *Fn1,* as well as genotype-specific differences (Figure 2A), in line with the notion that the identified profibrotic phenotype is not fully captured by the traditional M1/M2 paradigm. Interestingly, LPS stimulation increased *Spp1* expression, with significantly higher *Spp1* expression in *Cxcl4*^*−/−*^ CD11b^+^ monocytes.

### *Cxcl4*^*−/−*^ mice are protected from fibrosis after organ injury

To next examine whether CXCL4-driven *Spp1*^*+*^ macrophage activation impacts fibrogenesis, we subjected *Cxcl4*^*−/−*^ and WT control mice to MI or sham surgery (Figure 2B). Histopathological analysis and automated quantification of the fibrosis-specific picrosirius red stain confirmed that *Cxcl4*^*−/−*^ mice developed significantly less fibrosis after MI (Figures 2C and 2D) as well as reduced MI scar sizes (Figure 2E). Most importantly, loss of *Cxcl4* preserved left ventricular ejection fraction (μ = 43.29%) after MI in comparison with WT control animals (μ = 31.38%) (Figure 2F, Table S2). Next, we set out to investigate whether loss of *Cxcl4* ameliorated fibrosis across organs. We therefore performed renal unilateral ischemia-reperfusion injury (IRI) in *Cxcl4*^*−/−*^ and WT mice (Figure 2G). Again, automated histopathological analysis confirmed that knockout of *Cxcl4* significantly decreased cortical fibrosis compared with WT control mice (Figures 2H and 2I).

### snRNA-seq of WT and *Cxcl4*^*−/−*^ mice after ischemic kidney injury

**Figure 3:**
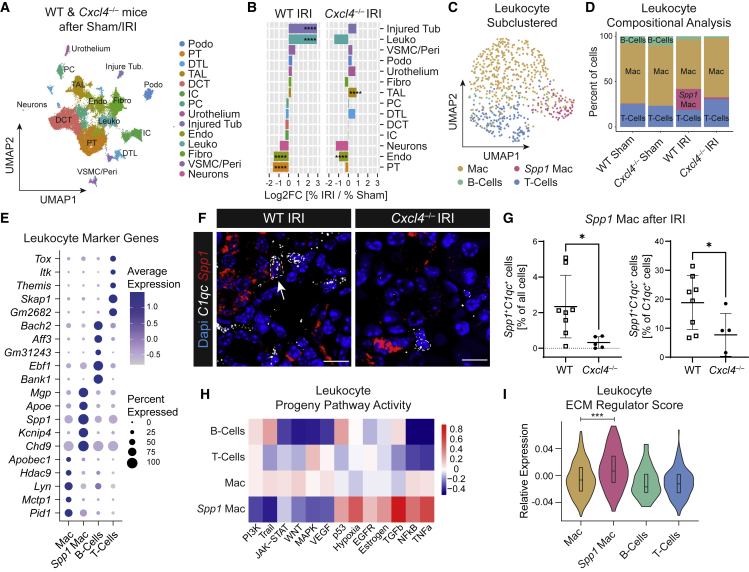
Loss of *Cxcl4* abrogates profibrotic *Spp1*^*+*^ macrophage expansion (A) UMAP embedding of 66,235 nuclei isolated from kidneys of WT and *Cxcl4*^*−/−*^ mice after sham or IRI surgery (n = 1 snRNA-seq library per condition pooled from n = 5 WT Sham, n = 5 WT IRI, n = 4 *Cxcl4*^*−/−*^ Sham, and n = 4 *Cxcl4*^*−/−*^ IRI mice). Labels refer to clusters. DCT, distal convoluted tubule; DTL, descending thin limb; Endo, endothelial cells; Fibro, fibroblasts; IC, intercalated cells; Injured Tub, injured tubular cells; Leuko, leukocytes; PC, principal cells; Podo, podocytes; PT, proximal tubule; TAL, thick ascending limb; Peri, pericytes; VSMC, vascular smooth muscle cells. (B) Bar plot of cluster cell numbers in IRI versus sham kidneys for WT and *Cxcl4*^*−/−*^ mice after normalization via Log2 transformation. Log2FC, log 2-Fold Change. (C) UMAP of 489 sub-clustered single cell leukocytes from (A). (D) Leukocyte sub-cluster composition (in % of all leukocytes) stratified by genotype and surgery. (E) Dotplot of the top five specific genes for leukocyte clusters shown in (C). (F) RNA-ISH staining for *C1qc* (white) and *Spp1* (red) in WT and *Cxcl4*^*−/−*^ kidneys after IRI. A white arrow marks a Spp1^+^C1qc^+^ cell (WT mice = 8, *Cxcl4*^*−/−*^ mice = 5). Scale bar =10 μm. (G) Quantification of *C1qc*^*+*^*Spp1*^*+*^ double-positive nuclei in percent of all nuclei and of *C1qc*^*+*^ nuclei. Data are shown as mean ± SD. (H) PROGENy pathway analysis of snRNA leukocyte clusters. (I) Leukocyte ECM regulator score stratified by leukocyte sub-cluster. For (B) Fisher’s exact test was computed using false discovery rate correction for multiple testing. For (G) and (I) a two-tailed unpaired t test was performed. ^∗^p < 0.05, ^∗∗∗^p < 0.001, ^∗∗∗∗^p < 0.0001. See also Figures S3, S4, and S6.

To dissect the molecular mechanisms and pathways through which CXCL4 propagates organ fibrosis, we performed snRNA-seq of murine WT and *Cxcl4*^*−/−*^ kidneys after IRI or sham surgery (Figure S3A). After quality control, doublet exclusion (Figure S3B) and data integration, clustering, and annotation demonstrated presence of all kidney cell populations (Figures 3A, S3C, and S3D, Table S3). Reference mapping of snRNA-seq data using Symphony to a published murine snRNA-seq time course of renal IRI^41^ corroborated our cluster annotation (Figures S3E and S3F). Compositional analysis by comparison of WT or *Cxcl4*^*−/−*^ IRI to sham kidneys confirmed strong injury in WT mice marked by loss of proximal tubular (PT) and endothelial cells (Endo) with concomitant expansion of injured tubular cells (Injured Tub) and leukocytes (Leuko) (Figure 3B). In contrast, loss of *Cxcl4* mitigated both loss of PT as well as expansion of injured tubular cells and leukocytes. As snRNA-seq compositional analysis compares only relative cell numbers, we validated major findings using an orthogonal method. Immunofluorescence stainings confirmed an increase in the number of tubular cells co-expressing the injury marker KIM1^+^ with a concomitant loss of LTL^+^ PT cells in WT mice after injury, which was mitigated in *Cxcl4*^*−/−*^ mice (Figures S4A–S4C). Whereas snRNA-seq compositional analysis detected a non-significant expansion of fibroblasts after injury, PDGFRα staining confirmed a significant expansion of fibroblasts in WT mice after IRI, which was nearly abrogated in *Cxcl4*^*−/−*^ mice (Figures S4A and S4D). In contrast, while snRNA compositional analysis suggested an expansion of TAL cells in *Cxcl4*^*−/−*^ mice after IRI, staining of the TAL-specific protein uromodulin revealed no relevant differences in TAL cell abundance between WT and *Cxcl4*^*−/−*^ mice (Figures S4A and S4E). Last, staining of CD68 showed a strong expansion of myeloid cells after IRI, with a non-significant reduction in *Cxcl4*^*−/−*^ mice (Figures S4A and S4F). These results confirmed our findings of a protective effect of genetic *Cxcl4* deletion on organ function and fibrosis after injury.

### Loss of *Cxcl4* abrogates expansion of profibrotic *Spp1*^*+*^ macrophages after ischemic kidney injury

Next, we set out to investigate whether loss of *Cxcl4* abrogates profibrotic *Spp1*^*+*^ macrophage expansion after IRI. Sub-clustering of leukocytes identified two macrophage clusters (Figure 3C), of which one, *Spp1*^*+*^ macrophages, expanded exclusively in WT mice after IRI, while being absent in *Cxcl4*^*−/−*^ animals (Figure 3D). Differential expression analysis confirmed that *Spp1*^*+*^ macrophages were characterized by similar marker genes as the above-described cardiac *Spp1*^*+*^ macrophages with specific expression of *Spp1*, *Fn1*, as well as expression of resident macrophage marker genes, *Apoe* and *C1qa* (Figures 3E and S4G, Table S3). Of note, despite well-established *Arg1* expression in kidney immune cells,^21^ we did not detect any relevant *Arg1* gene expression across the entire snRNA-seq dataset (Figure S4H), most likely due to sparsity of snRNA-seq data. To verify our findings of diminished *Spp1*^*+*^ macrophage expansion in *Cxcl4*^*−/−*^ IRI kidneys, we co-stained *Spp1* with the resident macrophage marker *C1qc*^42^ by in-situ hybridization (ISH) (Figure 3F). Automated quantification confirmed both overall loss of *Spp1*^*+*^ macrophages (Spp1^*+*^C1qc^*+*^ cells in % of total cells), and more importantly loss of *Spp1* expression in C1qc^*+*^ macrophages (Spp1^*+*^C1qc^*+*^ cells in % of C1qc^*+*^ cells) in *Cxcl4*^*−/−*^ mice after IRI (Figure 3G). To better characterize immune cell states, we inferred pathway activity using PROGENy. Similar to the previously identified cardiac *Spp1*^*+*^ macrophage cluster, kidney *Spp1*^*+*^ macrophages were characterized by high TGFβ and hypoxia pathway activity, coupled with latent proinflammatory NF-κB and TNF signaling. In contrast, the macrophage cluster Mac was defined by enrichment of proinflammatory pathways such as JAK-Stat, TNF, and NF-κB signaling, as well as anti-inflammatory pathways including vascular endothelial growth factor, and WNT signaling (Figure 3H). Analysis of the expression of inflammatory (*Irf2*, *Ccr2,* or *Nlrp1b*) and anti-inflammatory macrophage genes (*Trem2*) suggested that this cluster consists of a heterogeneous population of both infiltrating and resident macrophages (Figure S4I). In line with our initial finding, ECM regulator scoring of leukocyte sub-clusters confirmed highest ECM regulator scores in *Spp1*^*+*^ macrophages (Figure 3I). These findings confirm CXCL4 to be crucial for profibrotic *Spp1*^*+*^ macrophage expansion after injury.

### Platelets induce a profibrotic *Spp1*^*+*^ macrophage signature via CXCL4

**Figure 4:**
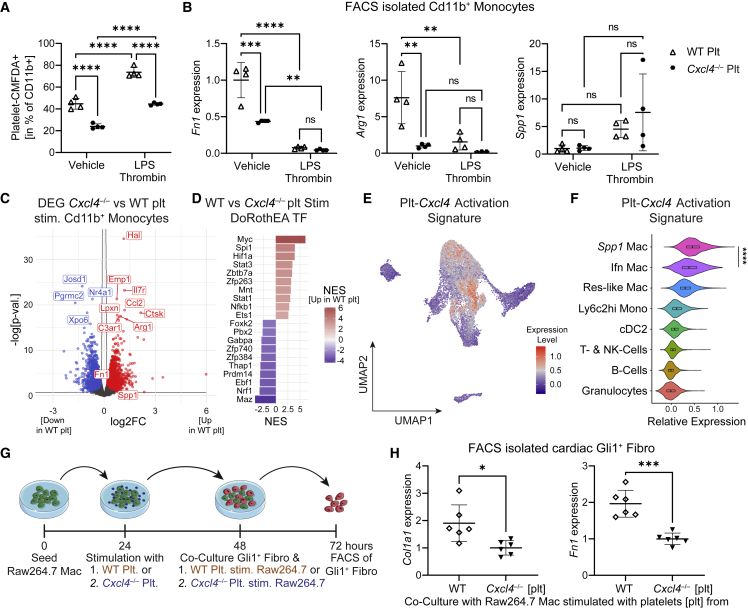
Platelet-derived CXCL4 drives profibrotic *Spp1*^*+*^ macrophage activation (A) Flow cytometric analysis of Platelet-CMFDA^+^CD11b^+^ platelet-monocyte aggregates after co-culture of WT PBMC with either CMFDA-positive WT or *Cxcl4*^*−/−*^ platelets and stimulation with Vehicle or LPS and Thrombin (n = 4). (B) RT-qPCR analysis for *Arg1*, *Fn1,* and *Spp1* in sorted CD11b^+^ monocytes after WT or *Cxcl4*^*−/−*^ platelet-induced activation of WT PBMC. Plt, platelets. (C) Volcano plot showing differentially expressed genes in CD11b^+^ monocytes activated with either WT or *Cxcl4*^*−/−*^ platelets (n = 4). p-val., p value; plt, platelets; stim., stimulated. (D) DoRothEA transcription factor analysis of differentially expressed genes in CD11b^+^ monocytes co-cultured with either WT or *Cxcl4*^*−/−*^ platelets. (E) Expression of a platelet-*Cxcl4* activation signature (top upregulated genes defined by an adjusted p value <0.01 and log2FC > 0.5 in WT versus *Cxcl4*^*−/−*^ co-cultured CD11b^+^ monocytes) in cardiac immune cells plotted on the UMAP embedding shown in Figure 1A. (F) Platelet-*Cxcl4* activation signature in cardiac immune cells stratified by immune cell type. (G) Experimental design of Gli1^+^-fibroblast co-culture with WT or *Cxcl4*^*−/−*^ platelet-stimulated Raw264.7 macrophages. Mac, macrophages; Fibro, fibroblasts; stim, stimulated. (H) RT-qPCR analysis of *Col1a1* and *Fn1* expression in Gli1^+^ cardiac fibroblasts after co-culture with WT or *Cxcl4*^*−/−*^ platelet pretreated Raw264.7 macrophages as shown in (C) (n = 6). For (A) and (B), a two-way ANOVA was computed using Tukey corrections. For (F) and (H), a two-tailed unpaired t test was performed. ^∗^p < 0.05, ^∗∗^p < 0.01, ^∗∗∗^p < 0.001, ^∗∗∗∗^p < 0.0001. See also Figure S5.

*In vivo*, activated platelets are considered the most abundant source of CXCL4, as they contain around 20 μg of CXCL4 per 10^9^ platelets, while monocytes only secrete comparatively minor amounts (approximately 0.1 μg/mL).^43^ We therefore questioned whether platelets are able to effectuate the same profibrotic *Spp1*^*+*^ macrophage signature. To analyze platelet-induced macrophage activation, we isolated platelets from WT and *Cxcl4*^*−/−*^ mice, labeled them with a live cell dye (CMFDA) and subsequently co-cultured them with WT peripheral blood mononuclear cells (PBMCs) in the absence or presence of LPS and thrombin. Twenty-four hours after platelet-PBMC co-culture, CD11b^+^ monocytes were isolated via fluorescence-activated cell sorting (FACS) (Figure S5A). Surprisingly, FACS revealed that *Cxcl4-*proficient platelets interact significantly more with CD11b^+^ monocytes as determined by quantification of CMFDA^+^CD11b^+^ cells (Figures 4A and S5B). Activation of platelets and monocytes with thrombin and LPS further increased platelet-monocyte interaction, while retaining genotype-specific differences (Figure 4A). Similar to our previous findings, monocytes co-cultured with WT platelets displayed higher expression of *Fn1* and *Arg1* compared with *Cxcl4*^*−/−*^ platelet-treated monocytes, whereas we did not see a significant difference in *Spp1* expression at baseline (Figure 4B). Again, LPS/Thrombin stimulation decreased *Fn1* and *Arg1* expression and reduced genotype-specific differences (Figure 4B). To verify decreased platelet-monocyte interaction with loss of *Cxcl4*, we imaged CMTPX-stained (red live cell dye) WT PBMCs after 48 h of co-culture with either CMFDA-stained WT or *Cxcl4*^*−/−*^ platelets. Indeed, platelet-adhering monocytes showed stronger CMFDA fluorescent signal after stimulation with WT platelets in comparison with *Cxcl4*^*−/−*^ platelets (Figures S5C and S5D), indicating that CXCL4 not only drives profibrotic monocyte activation, but is also critical for platelet-monocyte interaction.

To fully capture platelet CXCL4-induced monocyte activation, we performed Bulk RNA sequencing of FACS-isolated CD11b^+^ monocytes after co-culture with WT or *Cxcl4*^*−/−*^ platelets as described above (Figure S5A). After quality control and principal-component analysis (Figures S5E and S5F), differential expression analysis confirmed upregulation of known profibrotic genes, such as *C3ar1* and *Ctsk*,^44^^,^^45^ in WT platelet-stimulated monocytes in comparison with *Cxcl4*^*−/−*^ platelet stimulation (Figure 4C, Table S4). Strikingly, imputing TF activity using DoRothEA revealed high congruence to the previously identified cardiac *Spp1*^*+*^ macrophage signature with Myc, Hif1a, and Spi1 being the top three transcription factors, whose activities were upregulated in WT platelet-stimulated monocytes (Figure 4D, Table S4). In addition, PROGENy pathway analysis corroborated increased activity of TNF and NF-κB signaling in WT platelet-stimulated monocytes (Figure S5G). Next, we asked whether the observed *in vitro* phenotype of platelet CXCL4 induced monocyte activation resembles the *in vivo* identified profibrotic *Spp1*^*+*^ macrophage signature. To this end, we generated a platelet CXCL4 activation signature based on the top upregulated genes (log2FC > 0.5, adjusted p value <0.01) in WT versus *Cxcl4*^*−/−*^ platelet-stimulated monocytes. Comparing the expression of this platelet CXCL4 activation signature in immune cells derived from cardiac scRNA-seq after MI^23^ confirmed strongest enrichment of a profibrotic platelet CXCL4 signature in *Spp1*^+^ macrophages, followed by Ifn macrophages (Figures 4E and 4F). These findings confirm that platelet CXCL4 drives a profibrotic *Spp1*-like macrophage activation.

In order to confirm that the identified macrophage phenotype defined by *Arg1*, *Fn1,* and *Spp1* expression drives fibrosis, we investigated whether CXCL4 proficient platelet-activated macrophages can activate and drive fibroblast ECM expression. First, we stimulated Raw264.7 macrophages with WT or *Cxcl4*^*−/−*^ platelets over 24 h to induce the aforementioned profibrotic phenotype (Figure 4G). After 24 h, platelets were removed via repeated washing and stimulated macrophages were transferred to a co-culture with CMTPX-labeled cardiac murine Gli1^+^ fibroblasts that we generated for this experiment using *Gli1CreER*^*t2*^;*tdTomato* mice, tamoxifen pulse, and SV40 Large T immortalization (Figure S5H). Of note, we have previously shown that these cells are critical for fibrosis in ontogenetically distinct organs including the heart and kidney.^3^ FACS isolation (Figure S5I) and RT-qPCR analysis of CMTPX^*+*^ Gli1^+^ fibroblasts confirmed that macrophages stimulated with WT platelets induced significantly higher expression of *Col1a1* and *Fn1* in Gli1^+^ fibroblasts in line with activation of these cells toward matrix-producing myofibroblasts (Figure 4H).

**Figure 5:**
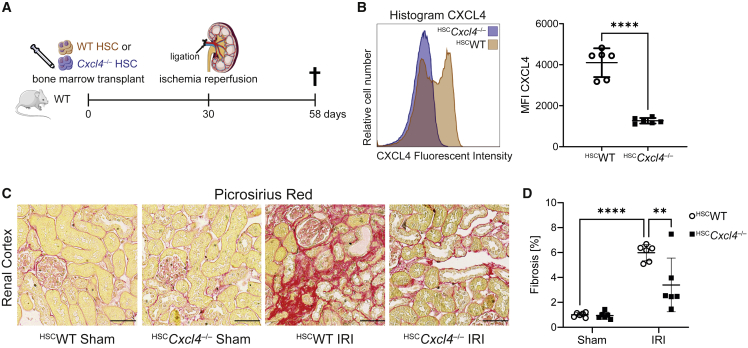
Hematopoietic *Cxcl4*^*−/−*^ mitigates kidney fibrosis after IRI (A) Experimental design for IRI surgery in mice after lethal irradiation and bone marrow transplantation with either WT (^HSC^WT) or *Cxcl4*^*−/−*^ (^HSC^*Cxcl4*^*−/−*^) hematopoietic stem cells. (B) Mean fluorescent CXCL4 intensity in peripheral blood of ^HSC^WT and ^HSC^*Cxcl4*^*−/−*^ mice 58 days transplantation (^HSC^WT mice = 6 and ^HSC^*Cxcl4*^*−/−*^ mice = 6). (C) Representative images of picrosirius red stained cortical kidney sections from ^HSC^WT and ^HSC^*Cxcl4*^*−/−*^ mice after sham or IRI surgery. Scale bar = 50 μm. (D) Kidney cortex fibrosis (in % of cortex area) after sham or IRI surgery by quantification of red ECM of scans shown in (G). All quantitative data are shown as mean ± SD. For (D) a two-way ANOVA was computed using Tukey corrections. For (B) a two-tailed unpaired t test was performed. ^∗∗^p < 0.01, ^∗∗∗∗^p < 0.0001. See also Figure S5.

To verify whether platelet- and monocyte-derived CXCL4 drives organ fibrosis *in vivo*, we generated a hematopoietic *Cxcl4* knockout by transplanting *Cxcl4*^*−/−*^ or WT hematopoietic stem cells (HSC) into lethally irradiated WT mice (Figure 5A). Thirty days after transplantation, mice were subjected to kidney unilateral IRI surgery with contralateral sham surgery. Based on our hypothesis, loss of *Cxcl4* in HSC and platelets should lead to a robust reduction in renal fibrosis. Flow cytometric analysis of CXCL4 in peripheral blood on day 58 validated knockout of *Cxcl4* (Figures 5B and S5J). Of note, lethal irradiation does not lead to complete loss of recipient HSC, and therefore a chimeric phenotype with residual CXCL4 expression by recipient HSC is expected. Automated quantification of ECM in a picrosirius red staining showed markedly less renal cortex fibrosis after IRI in animals that received *Cxcl4*^*−/−*^ bone marrow as compared with animals that received WT bone marrow (Figures 5C and 5D). This effect was similar to a complete loss of *Cxcl4* (≈43% reduction of renal fibrosis in ^HSC^*Cxcl4*^*−/−*^ mice compared with ≈52% reduction in *Cxcl4*^*−/−*^ mice compared with WT controls). Accordingly, RT-qPCR analysis demonstrated a strong reduction of *Col1a1* and *Fn1* expression in kidneys of ^HSC^*Cxcl4*^*−/−*^ mice after injury (Figure S5K), validating that hematopoietic-derived CXCL4 drives organ fibrosis after injury. In conclusion, we provide strong *in vitro* and *in vivo* evidence that platelet- and monocyte-derived CXCL4 drives organ fibrosis via profibrotic macrophage activation.

### Ligand-receptor interaction analysis confirms loss of macrophage-fibroblast crosstalk in *Cxcl4*^*−/−*^ mice after ischemic kidney injury

**Figure 6:**
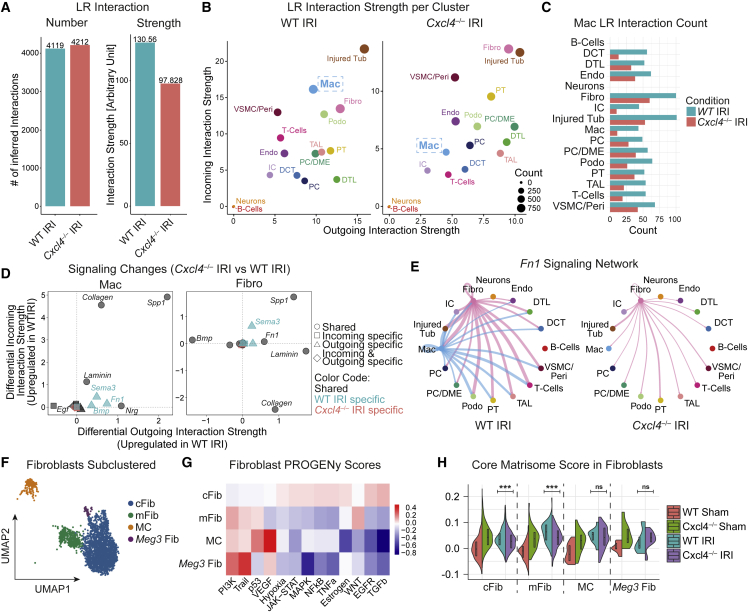
Loss of *Cxcl4* abrogates macrophage-fibroblast crosstalk (A) Total number of inferred ligand-receptor (LR) interactions and LR interaction strength for WT IRI and *Cxcl4*^*−/−*^ IRI kidneys. (B) Inferred outgoing and incoming LR interaction strength for individual clusters split by genotype. No LR interactions were found for neurons and B-cells. Labels refer to clusters. DCT, distal convoluted tubule; Endo, endothelial cells; Fibro, fibroblasts; IC, intercalated cells; Mac, macrophages; PC, principal cells; Peri, pericytes; Podo, podocytes; PT: proximal tubular cells; TAL, thick ascending limb; Tub, tubular cells; VSMC, vascular smooth muscle cells. (C) Inferred number of macrophage (Mac) Ligand-Receptor (LR) Interactions with cell clusters stratified by genotype. Labels refer to clusters. (D) Differential LR incoming and outgoing interaction strength (*Cxcl4*^*−/−*^ IRI versus WT IRI) by signaling network in macrophages (Mac) and fibroblasts (Fibro). (E) Network plots for inferred *Fn1* ligand-receptor interaction activity split by genotype. (F) UMAP of 2692 sub-clustered single nuclear fibroblasts from Figure 3A. cFib, cortical *Dapk2*^+^ fibroblasts; mFib, medullary *Dapk2*^−^ fibroblasts; MC, mesangial cells; *Meg3* Fib, *Meg3*^+^*Foxp2*^+^ fibroblasts. (G) PROGENy pathway analysis of fibroblast sub-clusters shown in (F). (H) Fibroblast Core Matrisome scores split by condition and fibroblast sub-clusters. For (H) a two-tailed unpaired t test was computed. ^∗∗∗^p < 0.001. See also Figure S6.

Although macrophages are key players in fibrosis, they do not represent a major cellular source of fibrosis-defining ECM (Figures S1E and S1F).^5^^,^^8^^,^^9^^,^^10^^,^^11^^,^^12^ Instead, macrophages are thought to orchestrate fibrogenesis indirectly via activation of ECM-secreting mesenchymal cells and modulation of ECM.^6^ To disentangle macrophage crosstalk, we performed CellChat Ligand-Receptor (LR) interaction analysis^46^ in the snRNA-seq datasets of WT IRI and *Cxcl4*^*−/−*^ IRI kidneys. To enable the analysis of macrophage crosstalk, we transferred cluster labels from the previously sub-clustered and annotated leukocytes to the integrated dataset containing all cell clusters. Crucially, as *Spp1*^*+*^ macrophages were nearly absent in *Cxcl4*^*−/−*^ IRI kidneys, we combined the two macrophage clusters, macrophages (Mac) and *Spp1*^*+*^ macrophages (*Spp1* Mac), into one macrophage cluster. Overall, LR interaction analysis identified a similar number of LR interactions across the two conditions, albeit with lower LR interaction strength in *Cxcl4*^*−/−*^ IRI kidneys (Figures 6A and S6A). This observation is in line with the notion of ongoing fibrotic remodeling in WT IRI kidneys. Plotting clusters based on both, their incoming and outgoing LR interaction strength, confirmed overall lower LR interaction strength in *Cxcl4*^*−/−*^ IRI kidneys (Figure 6B). Injured tubular cells (Injured Tub) and fibroblasts (Fibro) ranked among the top clusters for both WT and *Cxcl4*^*−/−*^ IRI kidneys in respect to LR interaction strength, underpinning their previously described central role in fibrosis. While macrophages in WT IRI kidneys displayed robust LR crosstalk (Incoming LR Rank 2, Outgoing LR Rank 8 with respect to WT IRI clusters), this crosstalk was abrogated in *Cxcl4*^*−/−*^ IRI kidneys (Incoming LR Rank 10, Outgoing LR Rank 13 with respect to *Cxcl4*^*−/−*^ IRI clusters).

Further investigation of Macrophage LR interaction count and strength indicated that macrophages primarily interact with injured tubular cells and fibroblasts (Figures 6C and S6B), with the latter cell type being the main cellular source of ECM in fibrosis. These LR interactions were dramatically reduced in terms of LR interaction count and interaction strength in *Cxcl4*^*−/−*^ IRI kidneys (Figure 6C). To identify the signaling networks via which macrophages may activate fibroblasts in WT animals after injury, we performed differential LR expression analysis (*Cxcl4*^*−/−*^ IRI versus *WT* IRI) for macrophages and fibroblasts (Figure 6D). Indeed, WT macrophages displayed higher outgoing LR interaction strength for known profibrotic ligand-receptor signaling networks, such as *Spp1*, *Fn1,* and *Sema3*, while WT fibroblasts showed higher incoming LR interaction strength for *Spp1* and *Sema3* (Figures 6D and S6C). Circular plots of *Fn1* (Figure 6E), *Spp1,* and *Sema3* networks (Figures S6D and S6E) confirmed strong macrophage-fibroblast crosstalk via these profibrotic networks in WT IRI, but not *Cxcl4*^*−/−*^ IRI kidneys.

### Loss of *Cxcl4* mitigates fibroblast activation after ischemic kidney injury

Based on LR interaction results, we hypothesized that loss of macrophage-fibroblast crosstalk mitigates subsequent fibroblast ECM expression. Sub-clustering of fibroblasts unmasked four distinct fibroblast sub-clusters, cortical *Dapk2*^+^ fibroblasts (cFib), medullary *Dapk2*^−^ fibroblasts (mFib),^41^^,^^47^ mesangial cells, and *Meg3*^+^ fibroblasts, which we previously identified as an undifferentiated fibroblast cell state^5^ (Figures 6F and S6F–S6H, Table S3). Corroborating our previous findings that interstitial fibroblasts represent the main cellular source of ECM in the kidney,^5^ inference of pathway activity using PROGENy confirmed high activity of the central profibrotic signaling pathways TGF^48^ and WNT^49^ in cortex and medullary fibroblasts, respectively (Figure 6G). Compositional analysis revealed no explicit differences in fibroblast composition across genotypes, with a trend for the enrichment of medullary fibroblasts in WT animals (Figure S6H). In contrast, scoring of fibroblasts based on their expression of core matrisome genes (core matrisome: collagens, proteoglycans, and glycoproteins) confirmed that loss of *Cxcl4* significantly reduced core matrisome expression in cortical (cFib) and medullary fibroblasts (mFib) after IRI (Figure 6H), consistent with a reduced activation of the fibrosis-driving fibroblast populations in *Cxcl4*^*−/−*^ mice.

### *SPP1*^*+*^ macrophages expand in chronic kidney disease and human heart failure

**Figure 7:**
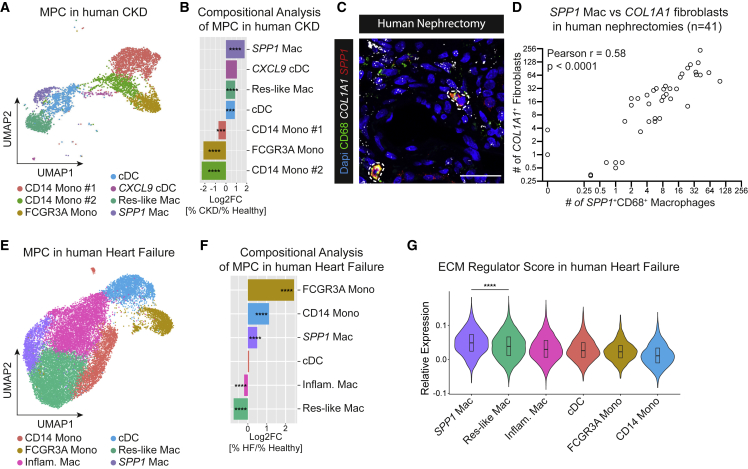
*SPP1*^*+*^ profibrotic macrophages expand in human CKD and heart failure (A) UMAP embedding of 4,404 mononuclear phagocytes sub-clustered from CD10^−^ single cells from 15 human kidneys by Kuppe et al.^6^ Labels refer to clusters. cDC, conventional dendritic cells; Mono, monocytes; Res-like Mac, resident-like macrophages; *SPP1* Mac, *SPP1*^*+*^ macrophages. (B) Bar plot of cluster cell numbers in CKD versus healthy kidneys after normalization via Log2 transformation. Log2FC, log 2-Fold Change. (C) RNA-ISH for *SPP1* and *COL1A1* combined with immunofluorescent CD68 staining in human kidney nephrectomies. *SPP1*^+^CD68^+^ macrophages are circled in white. Scale bar = 30 μm. (D) Pearson correlation of the number of *COL1A1*^+^ fibroblasts with *SPP1*^+^CD68^+^ macrophages in human kidney nephrectomies (n = 41). (E) UMAP embedding of 20,892 mononuclear phagocytes sub-clustered from CD45^+^ single cells from six human heart samples from Rao et al.^50^ Labels refer to clusters. Inflam. Mac, inflammatory macrophages. (F) Bar plot of cluster cell numbers in heart failure versus healthy hearts after normalization via Log2 transformation. Log2FC, log 2-Fold Change. (G) Cardiac ECM regulator score stratified by immune cell type. For (B) and (F), Fisher’s exact test was computed using false discovery rate correction for multiple testing. For (G), a two-tailed unpaired t test was performed. ^∗∗∗^p < 0.001, ^∗∗∗∗^p < 0.0001. See also Figure S7.

Last, to translate our findings to human disease, we sub-clustered MPC from our previously published scRNA-seq dataset of CD10 depleted (proximal tubule marker) cells isolated from seven healthy human kidneys (estimated glomerular filtration rate [eGFR] > 60 mL/min) and six kidneys with chronic kidney disease (CKD) due to hypertensive nephrosclerosis (eGFR <60 mL/min)^5^ (Figure S7A). After reclustering and annotation, we identified all major MPC populations (Figures 7A and S7B, Table S5). Analogous to our findings in the snRNA kidney and scRNA heart datasets, one macrophage cluster (*SPP1*^*+*^ macrophages) was defined by specific expression of *SPP1* and *APOE* (Figures S7B and S7C). Importantly, *SPP1*^*+*^ macrophages expanded more than any other MPC cluster in CKD kidneys (Figure 7B). As MPC numbers for individual kidney scRNA samples were low, we decided to verify our findings in a tissue microarray of 41 human kidneys. Combined ISH (*SPP1*, *COL1A1*) and immunofluorescence staining for the macrophage marker CD68 with subsequent quantification confirmed that *SPP1* expression in CD68 macrophages closely correlates with *COL1A1* expression in human kidneys (Figures 7C, 7D, and S7D).

To extend our insights to heart failure, we investigated a recently published immune cell scRNA-seq dataset of human heart failure^50^ (Figures S7E and S7F, Table S5). Sub-clustering of MPC from immune cells again identified a *SPP1*^*+*^ macrophage subset with specific expression of *SPP1*, *APOE*, and *FN1* (Figures 7E, S7G, and S7H, Table S5). Compositional analysis showed that monocytes accumulated most strongly, while *SPP1*^*+*^ macrophages were the only macrophage cluster to expand in failing hearts (Figure 7F). Similar to a recently described population of fibrosis-associated liver macrophages,^35^ *SPP1*^*+*^ macrophages specifically expressed *TREM2* and *CD9* in both CKD and heart failure (Figures S7I and S7J). Based on the overlapping marker profile, we next asked whether cardiac *SPP1*^*+*^ macrophages correspond to the identified kidney *SPP1*^+^ macrophages. Indeed, reference mapping of MPC from human heart failure to human CKD confirmed that cardiac *SPP1*^+^ macrophages best correspond to renal *SPP1*^+^ macrophages, highlighting a conserved phenotype of profibrotic macrophage activation across organs (Figures S7K and S7L). Finally, to investigate whether *SPP1*^*+*^ macrophages characterize a profibrotic macrophage subset in human heart failure, we again scored MPC based on their ECM regulator scores. Akin to our initial finding, human cardiac *SPP1*^*+*^ macrophages had the highest ECM regulator scores among all MPCs, highlighting their profibrotic potential in human disease (Figure 7G).

## Discussion

In this study, we uncovered a profibrotic macrophage population marked by expression of *Spp1*, *Fn1,* and *Arg1*. Using an unbiased approach, we identified and validated the chemokine CXCL4 to be essential for profibrotic macrophage activation and organ fibrosis. Mechanistically, we showed that platelets, the most abundant source of CXCL4 *in vivo*, control profibrotic macrophage activation via CXCL4. Translating our findings to human disease, we confirmed expansion of profibrotic *SPP1*^*+*^ macrophages in both human CKD and heart failure. Excitingly, LR interaction analysis uncovered macrophages as a central signaling hub in fibrotic kidneys that communicate with fibroblasts via potentially actionable LR networks (*Spp1*, *Fn1,* and *Sema3*).

In line with our findings of a profibrotic *SPP1*^*+*^ macrophage subset in kidneys and heart, Ramachandran et al. recently identified a fibrosis-associated macrophage population defined by *TREM2*, *CD9*, and *SPP1* expression in human livers.^35^ Independently, several recently published studies corroborated an *SPP1*^*+*^ macrophage subset in lung fibrosis.^20^^,^^51^^,^^52^^,^^53^ Both, identification of *SPP1*^*+*^ fibrosis-associated macrophages across organs, as well as loss of fibrosis after *in vivo* hematopoietic knockout of *Cxcl4* insinuate a monocytic origin for *SPP1*^*+*^ macrophages. Indeed, fine-mapping of MPC in metabolic fatty liver disease revealed that *Spp1* serves as a *bona fide* marker of monocyte-derived macrophages replacing resident Kupffer Cells after injury.^37^ A monocytic origin for *SPP1*^*+*^ macrophages would be consistent with several previous reports of aberrant monocyte-derived macrophages replacing pro-reparative resident macrophages after organ injury.^18^^,^^28^^,^^54^

Our work highlights CXCL4 as a critical mediator for *Spp1*^*+*^ macrophage differentiation and ultimately organ fibrosis. Previously, we and others have correlated CXCL4 with myelofibrosis and organ fibrosis.^55^^,^^56^^,^^57^ However, the exact mechanism by which CXCL4 exerts its profibrotic effect remains largely unexplored. Seminal work identified a key role for plasmacytoid dendritic cell (pDC)-derived CXCL4 in the pathogenesis of systemic sclerosis.^58^ Moreover, exogenous CXCL4 drove profibrotic activation of monocyte-derived dendritic cells *in vitro*.^59^ In contrast, we do not see any evidence for either *Cxcl4* expression or profibrotic activation of dendritic cells under pathophysiological conditions. We hypothesize that the reported differences are explained by the cellular source and time point of *Cxcl4* expression: In systemic sclerosis, continuous production of CXCL4 by pDC may drive capillary rarefaction, while in ischemic organ injury short-term local exposure to platelet- or monocyte-derived CXCL4 drives fibrosis via profibrotic macrophage activation.

Using PROGENy pathway analysis, we detected a latent proinflammatory activation with increased TNF signaling in *Spp1*^*+*^ macrophages. Interestingly, aberrant TNF signaling has been implicated in maladaptive tissue remodeling and fibrosis on multiple layers.^60^^,^^61^^,^^62^ While several studies have highlighted TNF as a central driver of inflammation and necroptosis during the acute phase of tissue damage,^63^ the subsequent role of TNF during tissue remodeling and fibrosis remains unclear. Notably, Dichtl et al. recently showed that TNF inhibits the emergence of a tissue reparative macrophage cell state by controlling a subset of canonical M2 genes.^64^ Reference mapping of *Spp1*^*+*^ macrophages onto a monocyte-derived framework of macrophage activation maps *Spp1*^*+*^ macrophages to an intermediate state of macrophage activation. Taken together, these findings imply that *Spp1*^*+*^ macrophages may represent immature tissue reparative macrophages that are trapped in an intermediate activation state due to perpetual TNF signaling. As such, TNF signaling might represent a central checkpoint in *Spp1*^*+*^ macrophage activation.

Physiologically, platelets are the first-line responders to vessel injury and are essential for hemostasis. Aside from their singular contribution to hemostasis, recent studies have increasingly recognized the indispensable role of platelets in leukocyte activation and function. As such, platelet-monocyte aggregates modulate viral infection,^65^ post-ischemic inflammation,^66^ and have been associated with severity and mortality of COVID-19.^67^ Intriguingly, platelets are able to shape macrophage polarization *in vivo,* both toward a proinflammatory^68^ or anti-inflammatory phenotype.^69^ However, evidence supporting a direct role for platelets in fibrosis remains weak. Here, we demonstrate that platelet-derived CXCL4 is critical for platelet-MPC interaction, subsequently driving profibrotic monocyte activation and fibrosis. These findings propose platelets as key regulators of organ remodeling by orchestrating monocyte activation, thus allowing platelets to control organ fibrosis beyond their short-lived nature. In light of these findings, targeting platelets to treat fibrosis may constitute an attractive therapeutic target.

In summary, we identify a profibrotic macrophage population defined by *Spp1*, *Fn1,* and *Arg1* expression. We demonstrated that *Spp1* profibrotic macrophage activation is dependent on CXCL4, which in return drove fibrotic tissue remodeling across organs. Strikingly, our findings provide an unexpected link between platelets, the main cellular source of CXCL4, macrophages, and fibrosis. Targeting platelet-macrophage interaction, potentially via inhibition of CXCL4, could serve as a springboard for innovative strategies aimed at mitigating organ fibrosis.

### Limitations of the study

It is important to note some limitations of our study. Despite detecting *Cxcl4* only in myeloid cells, *Cxcl4* expression was not limited to *Spp1*^+^ macrophages. Both resident-like and interferon macrophages also expressed *Cxcl4*, albeit at lower levels. We hypothesize that *Cxcl4* may mediate different functions depending on cell ontogeny and expression level. While we can demonstrate that loss of *Cxcl4* abrogates profibrotic activation of monocyte-derived macrophages both *in vitro* and *in vivo*, the effect of CXCL4 on resident macrophages remains yet to be elucidated. Another limitation of our study is the lower Unique Molecular Identifiers (UMI) and gene count of the *Cxcl4*^*−/−*^ sham library in comparison with other snRNA-seq libraries. To mitigate the impact of this potential technical bias, we integrated data using scVI and sub-clustered major cell types using Harmony while regressing out the impact of UMI counts and mitochondrial genes. To this end, core matrisome scoring suggested higher core matrisome scores in *Cxcl4*^*−/−*^ sham mice in comparison with WT sham mice. However, we confirmed no differences in kidney ECM deposition or gene expression in mice with complete or hematopoietic loss of *Cxcl4* after sham surgery (Figures 2H, 2I, 5C, 5D, and S5K). As such, this finding is likely a technical bias due to the differences in UMI and gene counts. Lastly, while we were able to show that loss of *Cxcl4* preserved cardiac function after injury, we did not assess kidney function after unilateral IRI, as the contralateral kidney has sufficient renal clearance to leave blood urea nitrogen and creatinine levels unaltered.

## STAR★Methods

### Key resources table

**Table:** 

REAGENT or RESOURCE	SOURCE	IDENTIFIER
**Antibodies**		

Anti-mouse CD11b (APC)	eBioscience	Cat# 17-0112-83; RRID: AB_469344
Anti-mouse mCD25 (PE-Cy7)	Biolegend	Cat# 102016; RRID: AB_312865
Anti-mouse CD4 (PB)	Biolegend	Cat# 100428; RRID: AB_493647
Anti-human CD68	Agilent	Cat# M0876; RRID: AB_2074844
Anti-mouse-Fc (AF488)	Jackson ImmunoResearch	Cat# 715-545-151; RRID: AB_2341099
mUromoduline	abcam	Cat# ab207170; RRID: AB_2889163
mPDGFRα	R&D Systems	Cat# AF1062; RRID: AB_2236897
LTL-Fitc	Vector Labs	Cat# FL1321; RRID: AB_2336559
mKIM1	R&D Systems	Cat#: AF1817; RRID: AB_2116446
mCD68	abcam	Cat# ab53444; RRID: AB_869007
Anti-rabbit-Fc (AF647)	Dianova	Cat# 111-605-008; RRID: AB_2338074
Anti-goat-Fc (Cy3)	Dianova	Cat# 705-165-147; RRID: AB_2307351
Anti-goat-Fc (AF647)	Dianova	Cat# 705-605-147; RRID: AB_2340437
Anti-rat-Fc (AF647)	Dianova	Cat# 712-605-153; RRID: AB_2340694

**Bacterial and virus strains**		

pBABE-puro SV40 LT vector	Addgene	#13970

**Biological samples**		

Human nephrectomy tissue samples (healthy and CKD)	This paper	N/A

**Chemicals, peptides, and recombinant proteins**		

LPS	Sigma-Aldrich	L4391-1MG
Thrombine	Molecular-Innovations	MTHROM-0.05MG
**Critical commercial assays**		

RNA-Scope™ Multiplex Fluorescent V2 Assay	ACD	323100
10x genomics single nuclear RNA-seq kit	10x genomics	1000077
Pikro Siriusred staining kit	Morphisto	13422
Cell Tracker CMFDA Dye	Thermo Fisher	C2925
Cell Tracker CMTPX Dye	Thermo Fisher	C34552
CD11b-Microbeads	Miltenyi Biotech	130-049-601
CD117-Microbeads	Miltenyi Biotech	130-091-224
High-Capacity cDNA Reverse Transcription Kit	Thermo Fisher	43-688-13
iTaq Univer SYBR Green Supermix	Biorad	1725125

**Deposited data**		

Data scRNAseq MI hearts (murine)	Forte et al.^23^	E-MTAB-7895
Data of human healthy and CKD kidney	Kuppe et al.^6^	10.5281/zenodo.4059315
Data of human heart samples	Rao et al.^50^	GSE145154
Reference mapping onto Dataset of Sanin et al.	Sanin et al.^38^	GSE171328, GSE157313
Reference mapping of snRNA-Seq Data of IRI kidney onto murine IRI-snRNA-Seq dataset	Kirita et al.^41^	GSE139107
Raw-Data snRNA-Seq IRI kidneys (WT + Cxcl4^−/−^)	This paper	ENA: PRJEB58150
Bulk-Sequencing Data of Monocyte-Platelet-Coculture	This paper	ENA: PRJEB58150
Original Code	This paper	https://github.com/KramannLab/Spp1MacFibrosis/

**Experimental models: Cell lines**		

Murine Cardiac Gli1+ cells	This paper	N/A

**Experimental models: Organisms/strains**		

*Cxcl4*^*一/一*^ (Black-Six-tm(*Cxcl4*)) mice	Gift from Wasmuth, Aachen	Zaldivar et al. 2010^56^
Gli1CreERt2 mice	Jackson Laboratories (Bar Harbor, ME, USA)	JAX Stock #007913
Rosa26tdTomato mice	Jackson Laboratories (Bar Harbor, ME, USA)	JAX Stock # 007909

**Oligonucleotides**		

RNA-Scope detection probe hCol1a1 (C1)	ACD	401891
RNA-Scope detection probe C2-hSPP1	ACD	420101-C2
RNA-Scope detection probe C2-mC1qc	ACD	496451-C2
RNA-Scope detection probe C3-mSPP1	ACD	435191-C3
For qPCR primers, see Table S6	N/A	N/A

**Software and algorithms**		

ImageJ	Schneider et al.^70^	imagej.nih.gov/ij/download.html
Ilastik	Berg et al.^71^	ilastik.org/download.html
RStudio Desktop		rstudio.com/products/rstudio/download/
GraphPad Prism version 9.0	N/A	graphpad.com/scientific-software/prism/
Scripts for image processing and analysis	This paper	https://github.com/thePowder/Cxcl4_image_analysis.git

**Other**		

8-well μ-Slides	Ibidi	80827

### Resource availability

#### Lead contact

Further information and requests for resources and reagents should be directed to and will be fulfilled by the lead contact, Rafael Kramann (rkramann@ukaachen.de).

#### Materials availability

For *in vitro* experiments we generated immortalized murine cardiac Gli1^+^ fibroblasts as described below. Immortalized murine cardiac Gli1^+^ fibroblasts are available from the lead contact with a completed Materials Transfer Agreement.

### Experimental model and subject details

#### Mice

*Cxcl4*^*−/−*^ (C57BL/6-tm(*Cxcl4*)) mice were obtained from Hermann Wasmuth (Aachen).^56^ Gli1CreER^t2^ (JAX Stock #007913) and Rosa26tdTomato (JAX Stock # 007909) were purchased from Jackson Laboratories (Bar Harbor, ME, USA). Genotyping was performed according to protocols from Jackson Laboratories. All animal protocols and procedures were approved by regional authorities (LANUV-NRW, Germany; Animal Welfare/Ethics committee of the EDC, Erasmus MC, Netherlands). 1 to 5 mice were kept in cages with unlimited admission to water and food on a 12-h light/dark cycle, room temperature at 20°C under specific-pathogen-free conditions. A gender- and age-matched design was chosen for all mouse experiments. The age of the mice ranged from 9 to 17 weeks. Further details about sex and age specifications can be found under the according paragraph under Methods details.

#### Human samples

The local ethics committee of the University Hospital RWTH Aachen approved all human tissue protocols (EK-016/17). Kidney tissue from non-tumorous human kidney was obtained from the Eschweiler/Aachen biobank. The age of the patients ranged from 30 years to 86 years with a mean of 66.22 years (standard deviation 12.97y; for details see Table S4, sheet 4). 14 women and 27 men were included. All patients gave informed consent, and the study was conducted in accordance with the Declaration of Helsinki.

#### Generation of immortalized murine cardiac Gli1^+^ fibroblasts

For induction of tdTomato expression *Gli1CreER*^*t2*^;*tdTomato* mice received three oral gavages with 10 mg Tamoxifen (Sigma-Aldrich) reconstituted in corn oil with 48 h between each gavage. 2 weeks after induction mice were sacrificed and cardiac Gli1; tdTomato^+^ fibroblasts were subsequently isolated via FACS of tdTomato-positive Gli1^+^ cells. 14 days after isolation and cell culture in DMEM medium (Thermo Fisher 31885), Gli1^+^ fibroblasts were immortalized by retroviral transduction of the pBABE-puro SV40 LT vector (Addgene #13970) as described previously.^5^ Three days after transduction, infected Gli1^+^ fibroblasts were selected using puromycin over a period of 7 days. Cells were cultured in DMEM (+10% FCS, +1% Penicillin/Streptomycin) and incubated in an CO_2_ incubator at 37°C with 5% CO_2_ gas supplement and >90% humidity.

#### Primary cells

##### Platelet isolation

Mice were anesthetized with isoflurane and killed by cervical dislocation. Subsequently, the thoracic cavity was opened and the right ventricle was punctured with a syringe, which was prefilled with 200 μL of sterile sodium citrate buffer. Blood was carefully aspirated and transferred into 2 mL tubes, prefilled with 250 μL acid-citrate-dextrose (ACD) buffer and 250 μL modified tyrode’s buffer (MTB). Samples were spun down in a swing-out centrifuge for 6 min at 200 G at room temperature (RT) with breaks turned off. The upper platelet rich plasma (PRP) was transferred while the remaining sample was washed again with 500 μL MTB, repeating the procedure. Collected PRP was spun down for 6 min at 600 G, supernatant discarded and platelets resuspended in media. Platelets were manually counted at least twice to adjust concentration between conditions (WT vs *Cxcl4*^*−/−*^) under a microscope using a Neubauer chamber and Trypan-Blue staining. Further incubation was carried out in DMEM (+10% FCS, +1% Penicillin/Streptomycin) in an CO_2_ incubator at 37°C with 5% CO_2_ gas supplement and >90% humidity.

##### PBMC isolation

Mice were anesthetized with isoflurane and killed by cervical dislocation. The thoracic cavity was opened, and blood was aspirated from the right ventricle with a syringe and transferred to EDTA tubes. Blood was mixed 1:1 with sterile PBS (1X, containing 2 mM EDTA) and layered onto Ficoll-Paque Plus (1.5-fold volume of blood, density = 1.077 g/mL). Density gradient centrifugation was performed for 40 min in a swing out centrifuge at 400 G with breaks turned off. Buffy coat layer containing PBMC was transferred, washed with PBS (1X, 2% FCS, 2 mM EDTA) and spun down for 10 min at 300 G. For platelet depletion, PBMCs were washed with PBS and spun down for 15 min at 200 G. Platelet contamination was evaluated by microscopy and cell-platelet ratios higher than 10 cells per platelet were considered as satisfactory. Cell pellets were resuspended in cell culture medium. Cells were cultured in RPMI-1640 (+10%FCS; +1% Penicillin/Streptomycin; 2 mM L-Glutamine) and further incubation was carried out in an CO_2_ incubator at 37°C with 5% CO_2_ gas supplement and >90% humidity.

### Method details

#### PBMC and platelet live cell imaging

PBMCs were collected from 2 WT mice (male). Platelets were isolated from 1 WT (male) and 1 *Cxcl4*^*−/−*^ mouse (male). WT PBMC were stained for 20 min with CMTPX CellTracker fluorescent dye (25 μM) in serum-free RPMI (containing 1% FCS, 2 mM L-Glutamine) at 37°C and washed with serum-free RPMI. WT and *Cxcl4*^*−/−*^ platelets were stained for 40 min with CMFDA CellTracker fluorescent dye (25 μM) in modified tyrodes buffer at 37°C for 40 min under smooth agitation and subsequently washed with modified tyrodes buffer. In an ibidi 8-well μ-slide, 1 × 10^5^ PBMCs and 7.5 × 10^6^ platelets were seeded in RPMI media (containing 10% FCS, 1% P/S, 2 mM L-Glutamine). After 48 h of incubation (37°C, 5% CO_2_) confocal laser microscopy was performed using a 100x objective as described below using 488 nm and 561 nm lasers. Z-stacks were preprocessed to obtain maximum-intensity projections with adjusted channel brightness using a script for ImageJ. A random forest classifier pipeline in Ilastik^71^ was used to perform pixel and object classification. In brief, adherent cells with lamellipodia in the CMTPX channel were recognized as monocytes/macrophages by their size, branching, intensity distribution and shape. By pixel classification specific cell located CMFDA signals were ranked by probability for positivity. Subsequent object classification revealed CMFDA-positive and CMFDA-negative objects by mapping beforementioned monocytes/macrophages with CMFDA probabilities.

#### PBMC and platelet Co-Culture with fluorescence-activated cell sorting (FACS)

PBMCs were isolated as described before from 8 WT mice. Platelets were isolated from 2 WT (male) and 2 *Cxcl4*^*−/−*^ (male) mice and stained with CMFDA (25 μM) as mentioned before. 6 × 10^5^ PBMC and 45 × 10^6^ WT or *Cxcl4*^*−/−*^ platelets were seeded into a 12-well plate (non-TC-treated) in 1 mL RPMI media (10% FCS, 1% P/S, 2 mM L-Glutamine). Stimulation group was stimulated with LPS (150 ng/mL) and Thrombin (4 IU/mL) while PBS was added to the control group as a vehicle. After incubation for 12 h (37°C, 5% CO_2_), samples were transferred into 2 mL tubes, remaining cells were trypsinized and conveyed to regarding samples. The suspensions were spun down, supernatant was aspirated and pellets were washed with PBS (2% FCS). Subsequently samples were stained with Anti-mCD11b (APC, 1:100, eBioscience, 17-0112-83), Anti-mCD25 (PE-Cy7, 1:100, Biolegend, 102016) and Anti-mCD4 (PB, 1:100, Biolegend, 100428) for 30 min on ice. Cell suspension was washed twice with PBS (+2% FCS) and 7-AAD (1:100) was added 5 min before sorting. Samples were sorted using a BD FACSMelody Cell sorter. Cells were gated by FSC, SSC and 7-AAD^-^ to obtain living single cells as parent and CD11b^+^ monocytes were sorted into RLT lysis buffer + 1% β-Mercaptoethanol for RNA isolation. To quantify monocyte platelet aggregates, CD11b^+^CMFDA^+^ monocytes were analyzed in comparison to total CD11b^+^ monocytes.

#### Gli-cell coculture with WT/Cxcl4^−/−^ platelet-activated monocytes

RAW264.7 cells were starved for 4 h with starving media (DMEM, no FCS, 1%P/S, GlutaMax). Platelets were isolated from 2 WT (male) and 2 *Cxcl4*^*−/−*^ (male) mice as aforementioned. RAW264.7 cells were cocultured with platelets (ratio cells:platelets = 1:100) from WT- or *Cxcl4*^*−/−*^ mice in 5 mL starving media (DMEM, no FCS, 1% P/S, GlutaMAX) in a T25 cell culture flask for 24 h. Murine cardiac Gli1^+^ cells were stained 20 min with 20 μM CMTPX (ThermoFisher, C34552) according to the manufacturer’s protocol and starved for 4 h using starving media (DMEM, no FCS, 1% P/S, GlutaMAX). Adhering Raw264.7 cells from platelet co-culture were washed, trypsinized and washed again. 3 × 10^5^ platelet-stimulated RAW264.7 cells were cocultured with 3 × 10^5^ starved cardiac Gli^+^ fibroblasts in a 6-well plate in 1mL DMEM (containing 1% FCS, 1% P/S, GlutaMAX). After 24 h cells were trypsinized, washed, stained with DAPI (1 μg/mL) and sorted into RLT-lysis buffer (containing 1% ꞵ-Mercaptoethanol) for CMTPX^+^DAPI^−^ single cells using a Sony SH800S Cell sorter. RNA was transcribed into cDNA and rt-qPCR was performed as described below.

#### Kidney ischemia-reperfusion injury

Male mice ranging from 9 to 12 weeks of age (8 WT and 5 *Cxcl4*^*−/−*^) were anesthetized by intraperitoneal injection (i.p.) with ketamine/xylazine (90 μg/g bodyweight (BW) ketamine, 9 μg/g BW xylazine). Analgesia was carried out by subcutaneous (s.c.) injection of metamizol (200 μg/g BW). For IRI procedure kidneys were exposed and mobilized by dorsolateral incision and perfusion was interrupted by clamping the renal artery using a non-traumatic microaneurysm clamp. Mice were kept at 37°C. After 28 minutes (min) ischemia, clamps were removed, and reperfusion was observed. The abdominal cavity was closed by peritoneal suture with prolene (6–0) and the skin clipped. For the sham procedure, skin and peritoneum were incised on the contralateral side, the kidney mobilized but not clamped, and the peritoneum and skin closed as described before. To minimize pain, metamizole (1.25 mg/mL) and 1% sucrose were added for three days to the drinking water. 28 days after IRI, mice were killed by cardiac puncture under ketamine/xylazine narcosis. The right ventricle was incised, the mouse was perfused with 30 mL PBS via the left ventricle and organs were taken for further analysis.^3^

#### Myocardial infarction

11 to 17-week-old sex- and age-matched WT (MI: 5x female, 3x male; sham: 5x female, 3x male) and *Cxcl4*^*−/−*^ (MI: 4x female, 3x male; sham: 4x female, 2x male) mice were subjected to myocardial infarction, as previously described.^72^ In brief, mice were anesthetized using isoflurane (2–2.5%), intubated and ventilated with oxygen using a mouse respirator (Harvard Apparatus, March, Germany). For analgesia, metamizole was injected subcutaneously (200 μg/g BW) in addition to local analgesia with subcutaneous and intercostal injection of Bupivacaine (2.5 μg/g BW). Left thoracotomy was performed and mice were subjected to sham surgery or myocardial infarction via ligation of the left anterior descending coronary artery (LAD) with a silk (0–7) suture. The ribs, muscle layer, and skin incision were closed using prolene (0–6), and metamizole was administered for three days via drinking water (1.25 mg/mL 1% sucrose) post-surgery.

#### Echocardiography

The left ventricular heart function was determined by echocardiography performed on a small-animal ultrasound imager (Vevo 3100 and MX550D transducer, FUJIFILM Visualsonics, Toronto, ON, Canada) 2 days before, as well as four and eight weeks after myocardial infarction. Measurements of short and long cardiac axes were taken in B-Mode (2D real-time) and M-Mode using a 40 MHz transducer (MX550D). During the procedure, mice were anesthetized with 1–2% isoflurane. Ejection fraction (EF), left ventricular end diastolic volume (LV-EDV), heart rate (HR) and left ventricular diameters were recorded and analyzed with VevoLab Software.

#### Bone marrow transplant experiment

11 host WT mice (male) ranging from 13 to 17 weeks of age were irradiated twice with 6.02 Gy in 4 h (Faxitron CP-160). 3 WT (male) and 3 *Cxcl4*^*−/−*^ (male) graft mice were sacrificed after isoflurane narcosis. Femora and tibiae were separated from muscle tissue and cleaned. Under sterile conditions, bone marrow was flushed out with syringes using phosphate buffered saline (PBS) with 2% fetal calf serum (FCS). Erythrocytes were lysed by incubating 5 min in 1x erythrocyte lysis buffer (BD Pharm Lyse) followed by two washing steps with PBS. Cell suspension was filtered through a 40 μm cell strainer. Tyrosine kinase KIT positive (cKIT+) cells were isolated by magnetic cell separation using murine cKIT-microbeads (Miltenyi Biotech) according to the manufacturer’s instructions. WT or *Cxcl4*^*−/−*^ cKIT^+^ stem cells were transplanted into the lethally irradiated host mice 2 h after the second radiation by retroorbital injection of 5 × 10^5^ cKit^+^ cells per mouse (6x WT, 5x *Cxcl4*^*−/−*^). To protect against infections during engraftment, antibiotics (Sulfadimethoxin & Trimethoprim, 95 mg/kg BW) were added to drinking water for 21 days after transplantation. 28 days after transplantation, success of hematopoietic stem cell engraftment was checked by taking blood samples and monitoring blood count. Mice were subjected to IRI or sham surgery as described before.

#### RNA ISH and immunofluorescence

RNA ISH was performed with the RNA-Scope detection kit (Multiplex Fluorescent Reagent Kit v2 Assay). The staining procedure was performed according to the manufacturer’s protocol for formalin fixed, paraffin embedded (FFPE) sections. We performed target retrieval for 30 min at 99°C and reduced the protease-treatment to 5 min. For RNA-hybridization the following probes were used: C1-hCol1a1 (401891), C2-hSPP1 (420101-C2), C2-mC1qc (496451-C2), C3-mSPP1 (435191-C3). If additional immunofluorescence staining was performed, tissues were blocked after RNA-ISH with 10% BSA for 30 min and incubated for 1 h at room temperature with primary antibody. After washing (3 × 5 min), slices were incubated with secondary antibodies for 30 min at room temperature. After washing (3 × 5 min) slices were counterstained with DAPI (1 μg/mL) for 2 min. Sections were mounted with ImmuMount mounting media and covered with coverslips. Used antibodies and dilutions are listed in Table S6.

#### ISH image analysis

Hybridized slides were imaged in a blinded fashion with a 60x Nikon objective and Z-stacks were acquired by local randomization at a resolution of 1024×1024 pixels^2^ using a Nikon A1R confocal laser microscope with wavelengths of 405 nm, 488 nm, 564 nm and 647 nm. The Z-stacks were computationally processed as a batch running a self-written script for ImageJ containing maximum intensity projection tool and brightness tool. A random forest classifier (Ilastik^71^) was trained and used to perform pixel and object classification.

#### Histology and Pathohistological examination

PBS perfused organs were fixed for 24 h in 4% paraformaldehyde (PFA) in PBS and embedded in paraffin. For heart tissue we performed serial slicing (10 levels, 200 μm apart) in line with the heart axis. 1 μm sections were deparaffinized and rehydrated using xylene and a descending row of ethanol. Picrosirius red (PSR) staining was performed using a PSR staining kit (Morphisto) following the manufacturer’s instructions. In brief, nuclei were stained for 8 min with Weigerts Eisenhämatoxylin, ECM was stained for 1 h in picrosirius red. Slides were washed with acetic acid and dehydrated in an ascending row of ethanol and xylene. The whole slide was imaged with a 40x objective using an Aperio AT2 Slide Scanner (Leica Biosystems). Images were analyzed using the Aperio ImageScope software by a blinded pathologist. Sirius-Red positive pixels were detected using a tissue classifier based on thresholding spectral intensity. For all slides the same threshold was used. Total fibrosis was defined as the ratio of picrosirius red positive pixels per total pixels of tissue. MI size was measured after manual assignment of the infarction area.

#### RNA extraction from tissue or cells

Snap frozen tissue samples were placed in RNAse free 2 mL tubes with 350 μL RLT lysis buffer with 1% β-Mercaptoethanol. After adding metal beads, the tissue was homogenized by shaking at 20 Hz for 2 min in a swing mill. Tissue lysates were spun down for 5 min at 300 G and RNA was isolated from the supernatant. For RNA isolation from cells, samples were incubated for 1 min in 350 μL RLT lysis buffer + 1% β-Mercaptoethanol. 350 μL supernatant or lysate were mixed with 350 μL 70% ethanol. RNA was isolated using the RNeasy Mini Kit (Qiagen) according to the manufacturer’s protocol. RNA was washed out in 15–35 μL RNAse free water.

#### Quantitative Real-Time PCR (qRT-PCR)

RNA concentration was measured using spectrophotometry and RNA-quality was estimated by 260nm:280nm and 260nm:230nm ratios. 300ng RNA was reversely transcribed to cDNA in 20 μL reaction volume using the High-Capacity cDNA Reverse Transcription Kit (Applied Biosystems). qRT-PCR was performed in duplicates using SYBR green master mix (Biorad) and the CFX Connect Real-Time System (Biorad). The cycle protocol was adjusted to 95°C for 2 min, 40 cycles of 10 s 95°C and 1 min 60°C followed by finally 5 s 95°C. *Gapdh* served as housekeeping gene. The 2-^ΔΔct^ was used for further gene expression analysis. Used primers are listed in Table S6.

#### Flow cytometry

Peripheral blood was sampled via retroorbital bleeding of isoflurane anesthetized mice. For red blood cell lysis, peripheral blood was incubated with a red blood cell lysis buffer (BD Pharm Lyse) for 10 min at room temperature before washing with PBS (1x, 2% FCS, 2 mM EDTA). Washed cells were filtered through a 70 μm cell strainer followed by labeling with a primary anti-CXCL4 antibody (R&D Systems, 1:100 in PBS, 2% FCS, 2 mM EDTA) for 30 min at 4°C. Subsequently, samples were washed three times with PBS (1x, 2% FCS, 2 mM EDTA) and conjugated with a secondary antibody (AF488, ThermoFisher, 1:200 in PBS, 2% FCS, 2 mM EDTA) for 15 min at 4°C. After conjugation, samples were again washed three times with PBS (2% FCS, 2 mM EDTA) before filtering through a 40 μm cell strainer to obtain single cell suspensions. Five minutes prior to data acquisition, 7AAD (eBioscience, 0.25 μg per 100 μL) was added for exclusion of non-viable cells. All samples were measured on a Sony SH800S Cell sorter and analyzed using FlowJo (v10.8, TreeStar Inc.).

#### Single nuclei isolation

Snap frozen kidney tissue was crushed using UV-irradiated, liquid nitrogen cooled porcelain mortars and subsequently homogenized in 500 μL nuclei lysis buffer (NLB, EZ lysis Buffer, NUC101, Sigma-Aldrich plus Protector RNAse Inhibitor, Roche) using glass tissue grind tubes and pestles. Homogenized solution was mixed with 4 mL NLB and filtered through a 40 μm cell strainer. Flowthrough was centrifuged at 500 G for 5 min at 4°C, supernatant discarded, and pellets were resuspended carefully in 5 mL of nuclei resuspension buffer (NRB). Suspension was spun down at 500 G for 5 min at 4°C. Supernatant was discarded and pellet was resuspended in 200 μL NRB. Five minutes prior to sorting DAPI (Sigma-Aldrich) was added to the suspension and subsequently DAPI^*+*^ nuclei sorted using a Sony SH800S Cell Sorter (Figure S3A).

#### 10x genomics (V3.1) single-nuclear assays

Sorted single nuclei were counted using a Neubauer Chamber and subsequently loaded onto a Chromium Next GEM Chip G Single Cell Kit (PN1-000120). Loading and cDNA library preparation was performed according to the manufacturer’s instructions (Chromium Next GEM Single Cell 3′ Kit v3.1). Library quality was assessed by D100 Screen Tape using a 2200 TapeStation system (Agilent Technologies). Libraries were sequenced on an Illumina NovaSeq system, targeting a read depth of 25000 reads/nuclei.

#### Bulk RNA library preparation

RNA was extracted from sorted CD11b^+^ monocytes as described above. cDNA and library construction were performed according to the manufacturer instruction using the NEBNext Single Cell/Low Input RNA Library Prep Kit (E6420L, Illumina). After assessment of library quality using the 2100 TapeStation system (Agilent Technologies), libraries were converted using the MGIeasy Universal Library Conversion Kit (1000004155). After conversion, library quality was assessed once more using the Agilent TapeStation system, before Libraries were sequenced on an DnbSeq-G400 system, targeting a read depth of 25000000 reads/library.

#### Single cell RNA (scRNA) data processing, quality control, cell filtering and batch-effect correction

Aligned public single cell datasets from Forte et al. and Rao et al. were obtained from ArrayExpress (E-MTAB-7895)^23^ and the Gene Expression Omnibus database (GSE145154).^50^ For analysis of MPC in human CKD we used our recently published, pre-filtered and annotated scRNA dataset of human CD10 negative kidney cells.^5^ All single cell datasets were analyzed with Seurat (v4.0) using custom scripts for pre-processing, quality control, data integration and differential expression analysis. Sample QC and data integration for all three datasets were performed according to the information provided by the authors in the original paper. For the dataset from Forte et al. all datasets (2x Sham, 1x each 1 day, 3 days, 5 days, 7 days, 14 days and 28 days after MI) were merged (*merge* function, Seurat) and cells with less than 500 features, more than 5000 features or more than 10% mitochondrial gene fraction removed. The latter was quantified by identification of mitochondrial gene names starting with “mt-” and quantification of “percent of mitochondrial gene-content per cell”. For the dataset from Rao et al. individual samples (1x Control with sampling of left and right ventricle, 2x DCM with sampling of left and right ventricle, 3x ICM with sampling of myocardial infarction and non-myocardial infarction tissue each) were integrated using a Harmony-based batch correction (v0.1.0).^73^ Of note, two samples (1x Control #2, 1x DCM #1) were not uploaded to the GEO database by the authors and could therefore not be included in the analysis. Subsequently all cells with an UMI Count of less than 800, more than 8000 or above 10% mitochondrial gene fraction were removed. Furthermore, genes that were present in less than 3 cells were filtered out.

After exclusion of low-quality cells, datasets were normalized (*NormalizeData* Function, Seurat), variable features identified (*FindVariableFeatures*, Seurat, features set to 2000) and scaled (*ScaleData*, Seurat). Principal components (*RunPCA*, Seurat) were calculated and plotted as Elbow Plots to individually determine optimal dimensions for UMAP representations. UMAPs were calculated (*RunUMAP*, Seurat) with dimensions set between 20 and 30 based on previous Elbow Plots. For Harmony-integrated datasets the PCA and UMAP representation were calculated based on the Harmony reduction.

#### scRNA clustering and cell-type annotation

Clustering (*FindNeighbors*, *FindClusters*, Seurat v4.0) and differential expression analysis based on clusters (*FindMarkers*, Seurat, for more information see below) was used to identify marker genes and manually annotate clusters based on information from literature.

#### scRNA cell type sub-clustering and further filtering

Sub-clustering of the major cell types was performed by subsetting the cell types of interest, and if Harmony-based batch correction was applied initially, re-calculating variable genes and Harmony-based batch correction with subsequent recalculation of Principal components. After re-calculating UMAP representations, reclustering and manual annotation of clusters based on marker genes was performed and contaminating clusters were removed (e.g. non-immune cells such as fibroblasts in sub-clustered immune cells from Forte et al.).

#### Single nuclear RNA (snRNA) data processing, quality control, cell filtering and batch-effect correction

After 10X sequencing, cellranger (v3.0.2), Scanpy (1.8.1), Seurat (v4.0) and custom scripts were used for preprocessing, quality control (QC) and gene-expression matrix generation. Additionally, Scrublet (0.2.3) was used for doublet detection. Reads from our single-cell experiment were demultiplexed using “*mkfastq*” and aligned to the mouse genome mm10 using “*count*” functions implemented in Cellranger (v3.0.2) run with default parameters. For each sample, Unique Molecular Identifiers (UMI) distribution was plotted and visually inspected to identify failed experiments. The samples that passed initial QC were aggregated and low-quality cells were removed. Only cells that had a gene count between 200 and 3000, less than 5% mitochondrial gene-content and a UMI count of less than 10000 were retained. Furthermore, genes that were present in less than 3 cells were filtered out. The final dataset consisted of 66369 cells and 24438 genes. To account for inter-sample biological heterogeneity, we utilized the single-cell variational inference (scVI) method (version 0.13.0) to map cells into a joint coordinate space. Here, sample id was used as the batch variable along with 2000 highly variable genes calculated using the “*highly_variable_gene*” method implemented in Scanpy (setting = seurat_v3) to calculate 50 latent variables.

#### snRNA clustering and cell-type annotation

The 50 latent variables obtained from scVI were used to cluster the dataset. We utilized the “*neighbors*” (n_neighbors = 50, distance metric = euclidean) function to calculate the neighborhood graph followed by “*leiden*'' clustering to identify 15 major cell types at resolution 0.5. The data was log normalized and marker genes were calculated using the “rank_genes_groups'' method in Scanpy. The clusters were manually annotated based on the marker genes.

#### snRNA cell type sub-clustering and further filtering

Sub-clustering of the major cell types was performed by using Harmony^73^ after subsetting the cell-type of interest. For each cell-type, 2000 highly variable genes were used to compute PCA (principal component analysis). The effect of the number of UMIs and mitochondrial gene-content was regressed out using the “*regress_out*” function implemented in Scanpy. Using 50 principle components and sample id as batch variable, Harmony was used to remove inter-sample batch-effects. Sub-clustering was performed using the Leiden algorithm in Scanpy. Sub-clusters were manually annotated based on the marker genes obtained from Scanpy’s “*rank_genes_groups*'' method and contaminating cell clusters removed (e.g. proximal tubular cells, in sub-clustered immune cells) after manual annotation.

#### Differential expression analysis

Differentially expressed genes between clusters were calculated using the “*FindAllMarkers”* function from Seurat (v4.0), using MAST run with the following settings: min.pct = 0.3. When plotting the expression of individual genes per cluster, the p values derived from the previously performed differential expression analysis using FindAllMarkers (MAST) were displayed.

#### ECM and platelet CXCL4 activation scores (functional analysis)

Scores were computed by using the function “*AddModuleScore*'' from Seurat (v4.0) at a single cell level. For ECM scoring, ECM gene sets defined by the matrisome project^24^ were used. For scoring of the platelet-*Cxcl4* activation signature, the top upregulated genes (adj. p value < 0.01 and log2FC > 0.5) derived from the comparison of CD11b^+^ monocytes co-cultured with WT vs *Cxcl4*^*−/−*^ platelets were used. For statistical comparison of Scores across conditions (e.g. WT IRI vs *Cxcl4*^*−/−*^ IRI) we computed statistical significance using an unpaired two-tailed T Test (stats, v4.1).

#### Trajectory inference analysis

PHATE embeddings were computed on a normalized matrix by setting parameters as 2 for ndim and 20 for decay using the package phateR (phateR version 1.0.7).^74^ Subsequently, Pseudotime was inferred by applying Slingshot (v2.2.0)^37^ on PHATE embeddings. Vector Generalized Linear and Additive Models^75^ implemented in the R package Monocle (v.2.21.1)^76^ were used to identify differentially expressed genes along the pseudotime. Linear smoothing approach provided by the VGAM was used for differentially expressed genes and visualization for the heatmap. Genes with a q-value of less than 0.01 were taken for further analysis.

#### ECM-regulator Score - Gene correlation analysis

For ECM Regulator - gene correlation analysis we followed the approach previously described by Iacono et al.^77^ Briefly, immune cells were subjected to clustering at a high-resolution to obtain a large number of transcriptionally homologous clusters. Gene expression and ECM regulator scores were averaged per cluster to mitigate the effect of a sparse matrix and improve correlation values. Finally, Pearson correlation was calculated based on average gene expression across clusters.

#### Cluster compositional analysis

For compositional analysis of clusters we applied Fisher’s exact test with Bonferroni correction. Cluster composition is displayed as bar plots of the log2-fold change of the normalized (to total cell count) cluster cell number in treatment [IRI/CKD/ICM/DCM] versus control [sham/healthy].

#### CellChat analysis (functional analysis)

Normalized data were used separately for each condition, but the cell types with less than 10 cells were filtered out. Intercellular communications between every two cell types were inferred by using the CellChat R package (v1.1.3).^46^ In a given ligand-receptor database provided by CellChat, paracrine/autocrine signaling interactions (“Secreted signaling”) and extracellular matrix (ECM)-receptor interactions (“ECM-receptor”) were selected for this study. Interaction strength is a measure of the communication probability between a given ligand-receptor interaction and is calculated as the degree of cooperativity/interactions derived by the law of mass action with the expression value of ligands and receptors.

#### Symphony reference mapping

Reference datasets for Sanin et al.,^38^ Kirita et al.,^41^ and Kuppe et al.^5^ were integrated with Harmony to create a new UMAP embedding that allows for Symphony reference-mapping. Cluster Annotations by the original authors were kept to allow for an unbiased reference-mapping. The datasets of macrophages after myocardial infarction,^23^ *Cxcl4*^*−/−*^ mice after Sham or IRI surgery, and MPC in human heart failure^50^ were mapped to the respective reference single-cell datasets with Symphony.^39^ For the annotation of the query samples with the reference labels, a k-NN classifier was applied. The percentage of cells of each query cell type are shown in the Figures.

#### Bulk RNA analysis

Preprocessing was performed according to the nf-core nextflow pipeline (version 21.04.1) using nf-core/rnaseq (version 3.1),^78^ star (version 2.7.9a) for read alignment,^79^ salmon (version 1.5.0) for read quantification,^80^ trimgalore (version 0.6.6) for read trimming, and gencode (version 38) for gene annotation.^81^ After generation of the count matrix file using salmon, mitochondrial and ribosomal genes (defined as “Mt_tRNA”, “rRNA”, “Mt_rRNA” or “rRNA_pseudogene” in the column gene_type) were filtered out, and subsequently low expressed genes were removed using HTSFilter (version 1.32.0).^82^ In addition, genes without a canonical gene name (starting with “Gm”) were also filtered out. After filtering, DESeq2 (version 1.32.0) was used to calculate differentially expressed genes from the filtered count matrix file.^83^ Finally PROGENy pathway activity and DoRothEA transcription factor activity were inferred as described below.

#### Pathway RespOnsive GENes (PROGENy) for activity inference analysis

For single-cell and single-nuclear RNA sequencing data we inferred PROGENy pathway activity using the murine version of PROGENy (version 1.16.0) based on the top 500 most responsive genes as recommended by a benchmark study.^26^^,^^30^^,^^84^ For analysis of bulk RNA sequencing data we inferred PROGENy pathway activity as previously described.^55^ In short, original pathway activity scores were inferred based on gene t-values obtained from DE-seq analysis. Repeated permutation (10,000x) of t-values was subsequently used to generate a null-distribution, and original pathway scores scaled to their respective null distribution to calculate a normalised pathway activity score.

#### DoRothEA transcription factor analysis

For inference of transcription factor activity in single cell RNA sequencing data, we used the murine version of DoRothEA (version 1.6.0), a collection of transcription factor targets, combined with VIPER (version 1.28.0) as recommended by a recent benchmark study.^29^^,^^30^^,^^84^ For Bulk RNA sequencing analysis, transcription factor activity was inferred from t-values obtained from DE-seq analysis using VIPER (version 1.28.0) as previously described.^55^ For both, single cell RNA and Bulk RNA sequencing analysis dorothea regulons with confidence levels A, B, and C were used.

### Quantification and statistical analysis

All data are shown as mean ± SD. Spearman’s rank correlation test for heteroscedasticity, Anderson-Darling and Shapiro-Wilk normality tests were performed to test for Gaussian distribution and homoscedasticity. To calculate differences between two-set sample data an unpaired two-tailed t test was performed. For statistical analysis of two-factor models, an ordinary two-way ANOVA with Tukey correction for multiple testing was computed. For correlation analysis, two-tailed nonparametric Spearman correlation was calculated. Graph-Pad Prism version 9 was used to perform statistical analysis. Given a p value < 0.05, a significant difference in distributions was considered.

## Data Availability

• Single nuclear RNA sequencing and bulk RNA sequencing data have been deposited at ENA and are accessible under the Project-ID ENA: PRJEB58150. All other accession numbers are listed in the key resources table. Microscopy data reported in this paper will be shared by the lead contact upon request. • All original code has been deposited at github and is available under the following link: https://github.com/KramannLab/Spp1MacFibrosis/. • Any additional information required to reanalyze the data reported in this paper is available from the lead contact upon request.

## Supplemental information

Document S1. Figures S1–S7

Table S1. scRNA-seq data of murine cardiac cells after myocardial infarction (Forte et al.) Sheet 1: Marker genes of cell clusters. Sheet 2: Marker genes of sub-clustered cardiac immune cells. Sheet 3: Differentially Expressed Genes along Slingshot Trajectory

Table S2. Echocardiographic measurements of WT and *Cxcl4*^*一/一*^ mice after sham or myocardial infarction (MI) surgery F, Female, M, male, EF, left ventricular ejection fraction, EDV, left ventricular end-diastolic volume

Table S3. snRNA-seq data of WT and *Cxcl4*^*一/一*^ mice after sham or ischemia-reperfusion injury Sheet 1: Marker genes of renal cell clusters. Sheet 2: Marker genes of sub-clustered leukocytes. Sheet 3: Marker genes of sub-clustered fibroblasts

Table S4. Bulk RNA-seq data of WT versus *Cxcl4*^*一/一*^ platelet co-cultured Cd11b + monocytes Sheet 1: Differentially expressed genes in monocytes. Sheet 2: DoRothEA transcription factor analysis based on differentially expressed genes

Table S5. Human scRNA-seq data Sheet 1: scRNA-seq marker genes of sub-clustered renal mononuclear phagocytes in human chronic kidney disease from Kuppe et al. Sheet 2: scRNA-seq marker genes of cardiac immune cells in human heart failure from Rao et al. Sheet 3: scRNA-seq marker genes of cardiac mononuclear phagocytes in human heart failure from Rao et al. Sheet 4: Sex and age of patients from whom human samples were taken for the TMA

Table S6. Antibodies and primers Sheet 1: Overview of used antibodies and dilutions for immune staining. Sheet 2: qPCR primers

Document S2. Article plus supplemental information
