# Supplementary material for: Platelet-instructed SPP1+ macrophages drive myofibroblast activation in fibrosis in a CXCL4-dependent manner
Source: Cell Rep. 2023 Feb 18;42(2):112131. doi: 10.1016/j.celrep.2023.112131 (PMC9992450; doi:10.1016/j.celrep.2023.112131)
Supplement: Document S1. Figures S1–S7 [file mmc1.pdf]

Cell Reports, Volume *42*

Supplemental information

Platelet-instructed SPP1^{+} macrophages drive myofibroblast activation in fibrosis in a CXCL4-dependent manner

Konrad Hoeft, Gideon J.L. Schaefer, Hyojin Kim, David Schumacher, Tore Bleckwehl, Qingqing Long, Barbara Mara Klinkhammer, Fabian Peisker, Lars Koch, James Nagai, Maurice Halder, Susanne Ziegler, Elisa Liehn, Christoph Kuppe, Jennifer Kranz, Sylvia Menzel, Ivan Costa, Adam Wahida, Peter Boor, Rebekka K. Schneider, Sikander Hayat, and Rafael Kramann

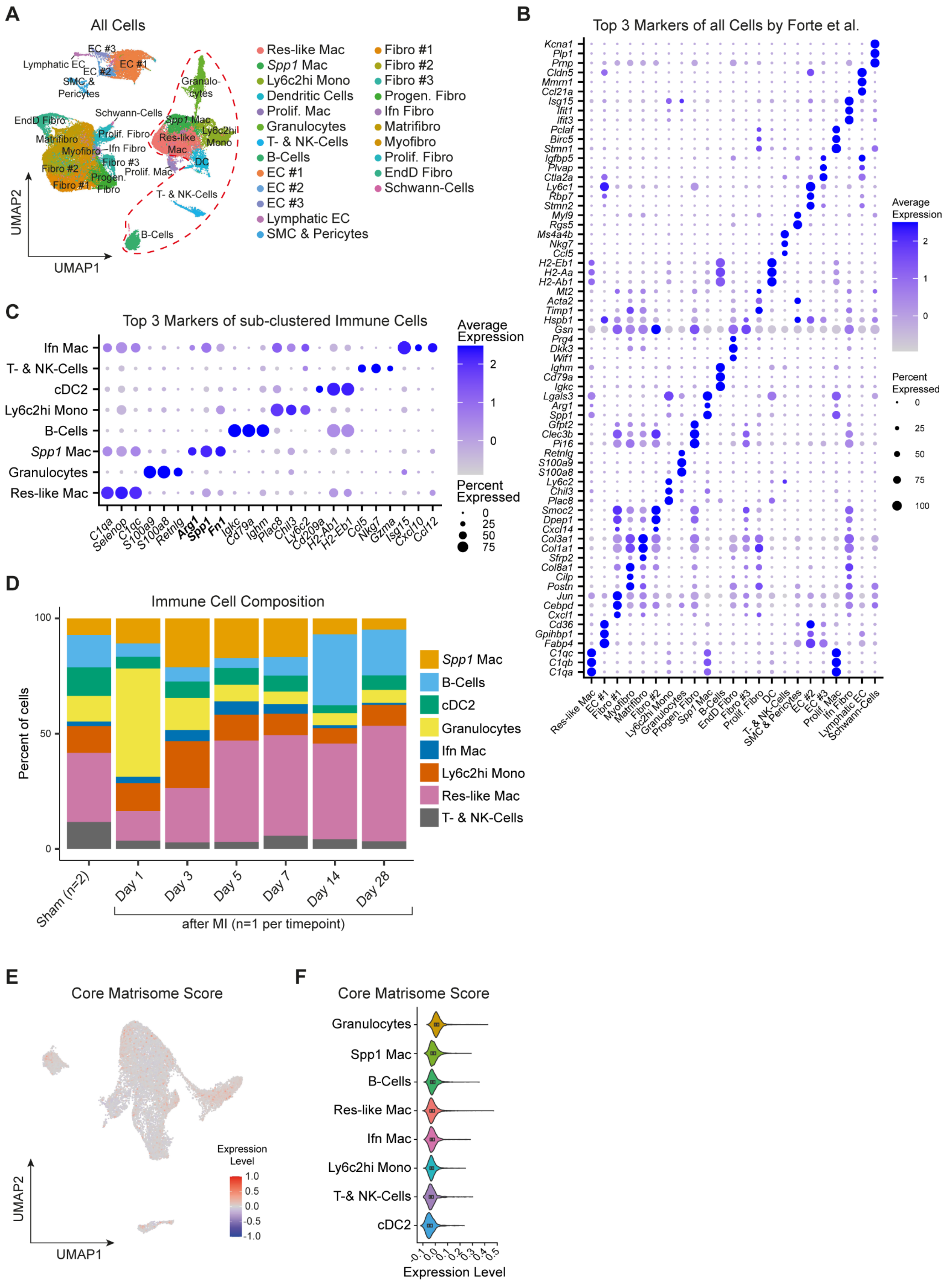
A
All Cells
UMAP1
UMAP2
EC #3
Lymphatic EC
EC #1
EC #2
SMC &
Pericytes
Granulo-
cytes
Schwann-Cells
Spp1 Mac
EndD Fibro
Matrifibro
Myofibro
Prolif. Fibro
Ifn Fibro
Fibro #3
Fibro #2
Fibro #1
Progen.
Fibro
Prolif. Mac
Res-like
Mac
Ly6c2hi
Mono
DC
T- & NK-Cells
B-Cells
Res-like Mac
Spp1 Mac
Ly6c2hi Mono
Dendritic Cells
Prolif. Mac
Granulocytes
T- & NK-Cells
B-Cells
EC #1
EC #2
EC #3
Lymphatic EC
SMC & Pericytes
Fibro #1
Fibro #2
Fibro #3
Progen. Fibro
Ifn Fibro
Matrifibro
Myofibro
Prolif. Fibro
EndD Fibro
Schwann-Cells
B
Top 3 Markers of all Cells by Forte et al.
Kcna1
Plp1
Prnp
Cldn5
Mmrn1
Ccl21a
Isg15
Ifit1
Ifit3
Pclaf
Birc5
Stmn1
Igfbp5
Plvap
Ctla2a
Ly6c1
Rbp7
Stmn2
Myl9
Rgs5
Ms4a4b
Nkg7
Ccl5
H2-Eb1
H2-Aa
H2-Ab1
Mt2
Acta2
Timp1
Hspb1
Gsn
Prg4
Dkk3
Wif1
Ighm
Cd79a
Igkc
Lgals3
Arg1
Spp1
Gfpt2
Clec3b
Pi16
Retnlg
S100a9
S100a8
Ly6c2
Chil3
Plac8
Smoc2
Dpep1
Cxcl14
Col3a1
Col1a1
Sfrp2
Col8a1
Cilp
Postn
Jun
Cebpd
Cxcl1
Cd36
Gpihbp1
Fabp4
C1qc
C1qb
C1qa
Res-like Mac
EC #1
Fibro #1
Myofibro
Matrifibro
Fibro #2
Ly6c2hi Mono
Granulocytes
Progen. Fibro
Spp1 Mac
B-Cells
EndD Fibro
Fibro #3
Prolif. Fibro
DC
T- & NK-Cells
SMC & Pericytes
EC #2
EC #3
Prolif. Mac
Ifn Fibro
Lymphatic EC
Schwann-Cells
Average
Expression
2
1
0
Percent
Expressed
0
25
50
75
100
C
Top 3 Markers of sub-clustered Immune Cells
Ifn Mac
T- & NK-Cells
cDC2
Ly6c2hi Mono
B-Cells
Spp1 Mac
Granulocytes
Res-like Mac
C1qa
Selenop
C1qc
S100a9
S100a8
Retnlg
Arg1
Spp1
Fn1
Igkc
Cd79a
Ighm
Plac8
Chil3
Ly6c2
Cd209a
H2-Ab1
H2-Eb1
Ccl5
Nkg7
Gzma
Isg15
Cxcl10
Ccl12
Average
Expression
2
1
0
Percent
Expressed
0
25
50
75
D
Immune Cell Composition
Percent of cells
100
50
0
Sham (n=2)
Day 1
Day 3
Day 5
Day 7
Day 14
Day 28
after MI (n=1 per timepoint)
Spp1 Mac
B-Cells
cDC2
Granulocytes
Ifn Mac
Ly6c2hi Mono
Res-like Mac
T- & NK-Cells
E
Core Matrisome Score
UMAP1
UMAP2
Expression
Level
1.0
0.5
0.0
-0.5
-1.0
F
Core Matrisome Score
Granulocytes
Spp1 Mac
B-Cells
Res-like Mac
Ifn Mac
Ly6c2hi Mono
T-& NK-Cells
cDC2
-0.1
0.0
0.1
0.2
0.3
0.4
0.5
Expression Level

Supplemental Figure 1: Single cell RNA analysis of a time-series of myocardial infarction. Related to Fig. 1.

A) UMAP embedding of all (52497) interstitial cells from murine heart tissue at different timepoints after MI from Forte et al., 2020. $Cd45^{+}$ immune cells (circled in red) were subsetted for further analysis. Labels refer to clusters. Res-like Mac: resident-like macrophages, *Spp1* Mac: *Spp1*$^{+}$ macrophages, Ly6c2hi Mono: Ly6c2hi monocytes, Prolif. Mac: Proliferating macrophages, T- & NK-cells: T-cells and natural killer cells, EC: Endothelial cells, SMC: Smooth muscle cells, Progen. Fibro: Progenitor-like fibroblasts, Ifn Fibro: Interferon-Induced fibroblasts, Prolif. Fibro: Proliferating fibroblasts, EndD. Fibro: Endocardial-derived fibroblasts. **B)** Dotplot of the top 3 specific genes for interstitial cell clusters shown in Fig. S1A. **C)** Dotplot of the top 3 specific genes for immune cell clusters shown in Fig. 1A. **D)** Bargraphs depicting the quantitative composition of immune cell subclusters shown in Fig. 1A **E)** Featureplot of the core matrisome score on the UMAP embedding shown in Fig. 1A. **F)** Core matrisome score stratified by immune cell type.

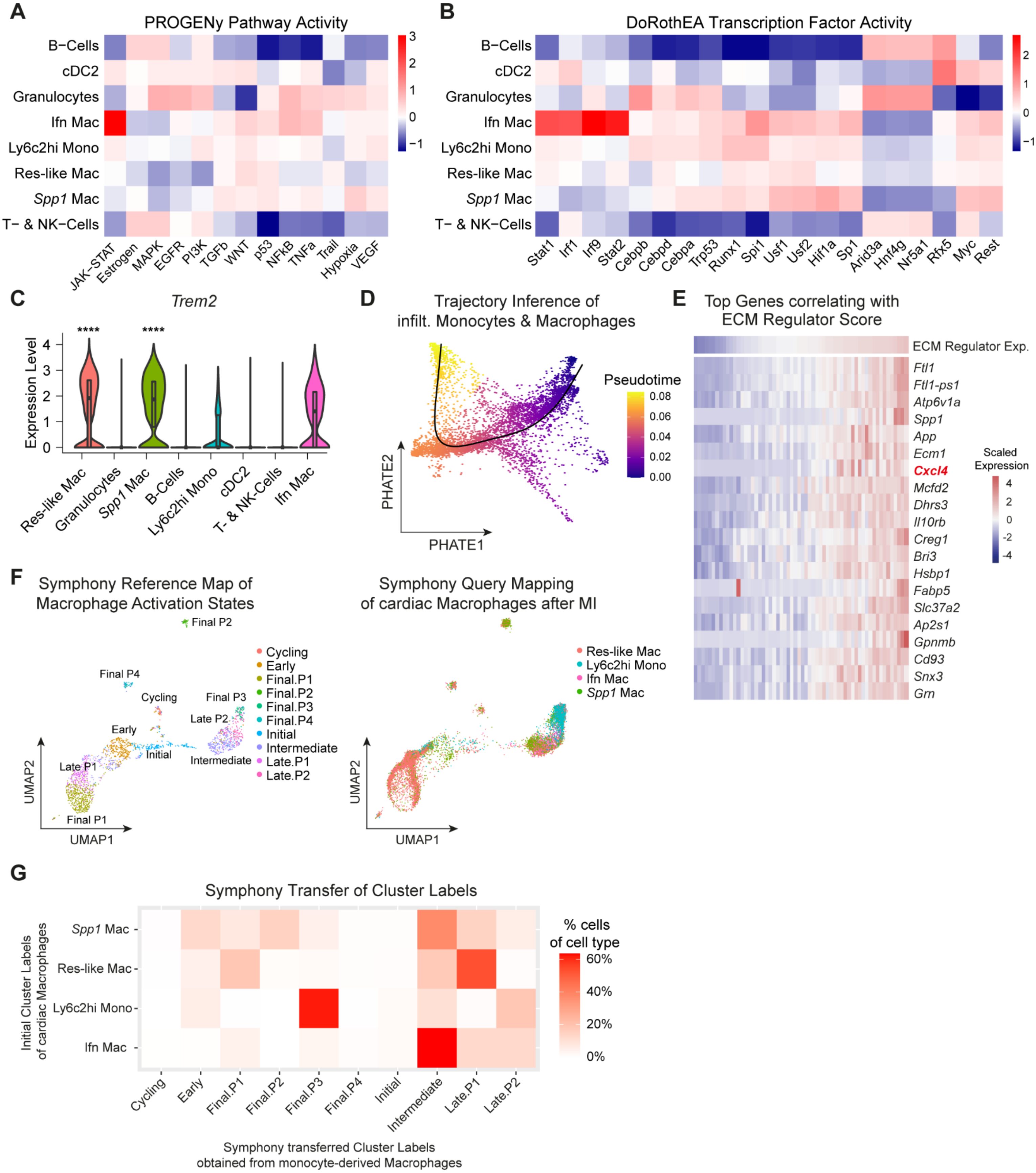
A
PROGENy Pathway Activity
B−Cells
cDC2
Granulocytes
Ifn Mac
Ly6c2hi Mono
Res-like Mac
Spp1 Mac
T− & NK−Cells
JAK-STAT
Estrogen
MAPK
EGFR
PI3K
TGFb
WNT
p53
NFkB
TNFa
Trail
Hypoxia
VEGF
3
2
1
0
−1
B
DoRothEA Transcription Factor Activity
Stat1
Irf1
Irf9
Stat2
Cebpb
Cebpd
Cebpa
Trp53
Runx1
Spi1
Usf1
Usf2
Hif1a
Sp1
Arid3a
Hnf4g
Nr5a1
Rfx5
Myc
Rest
2
1
0
−1
C
Trem2
Expression Level
****
****
Res-like Mac
Granulocytes
Spp1 Mac
B-Cells
Ly6c2hi Mono
cDC2
T- & NK-Cells
Ifn Mac
D
Trajectory Inference of
infilt. Monocytes & Macrophages
Pseudotime
0.08
0.06
0.04
0.02
0.00
PHATE1
PHATE2
E
Top Genes correlating with
ECM Regulator Score
ECM Regulator Exp.
Ftl1
Ftl1-ps1
Atp6v1a
Spp1
App
Ecm1
Cxcl4
Mcfd2
Dhrs3
Il10rb
Creg1
Bri3
Hsbp1
Fabp5
Slc37a2
Ap2s1
Gpnmb
Cd93
Snx3
Grn
Scaled
Expression
4
2
0
-2
-4
F
Symphony Reference Map of
Macrophage Activation States
Final P2
Final P4
Cycling
Final P3
Early
Late P2
Initial
Intermediate
Late P1
Final P1
Cycling
Early
Final.P1
Final.P2
Final.P3
Final.P4
Initial
Intermediate
Late.P1
Late.P2
UMAP1
UMAP2
Symphony Query Mapping
of cardiac Macrophages after MI
Res-like Mac
Ly6c2hi Mono
Ifn Mac
Spp1 Mac
UMAP1
UMAP2
G
Symphony Transfer of Cluster Labels
Initial Cluster Labels
of cardiac Macrophages
Spp1 Mac
Res-like Mac
Ly6c2hi Mono
Ifn Mac
Cycling
Early
Final.P1
Final.P2
Final.P3
Final.P4
Initial
Intermediate
Late.P1
Late.P2
Symphony transferred Cluster Labels
obtained from monocyte-derived Macrophages
% cells
of cell type
60%
40%
20%
0%

Supplemental Figure 2: Characterisation of cardiac *Spp1*$^{+}$ macrophages. Related to Fig. 1.

A) PROGENy pathway analysis of cardiac immune cell clusters shown in Fig. 1A. **B)** DoRothEA transcription factor analysis of cardiac immune cell clusters shown in Fig. 1A. **C)** *Trem2* expression stratified by immune cell clusters of 1A). **D)** Pseudotime Trajectory for infiltrating MPC on the PHATE embedding shown in Fig. 1D**. E)** Heatmap of the top genes correlating with ECM regulator scores in immune cells from Fig. 1A, *Cxcl4* is ranked #7. **F)** Symphony Reference Map of monocyte-derived macrophage activation states from Sanin et al. with Query Mapping of cardiac macrophages after myocardial infarction (MI) from Forte et al. into the same low dimensional space. **G)** Symphony Transfer of Cluster Labels of monocyte-derived activation states to cardiac macrophages. For C p-values from MAST (Seurat, FindAllMarkers) are displayed. ****P<0.0001.

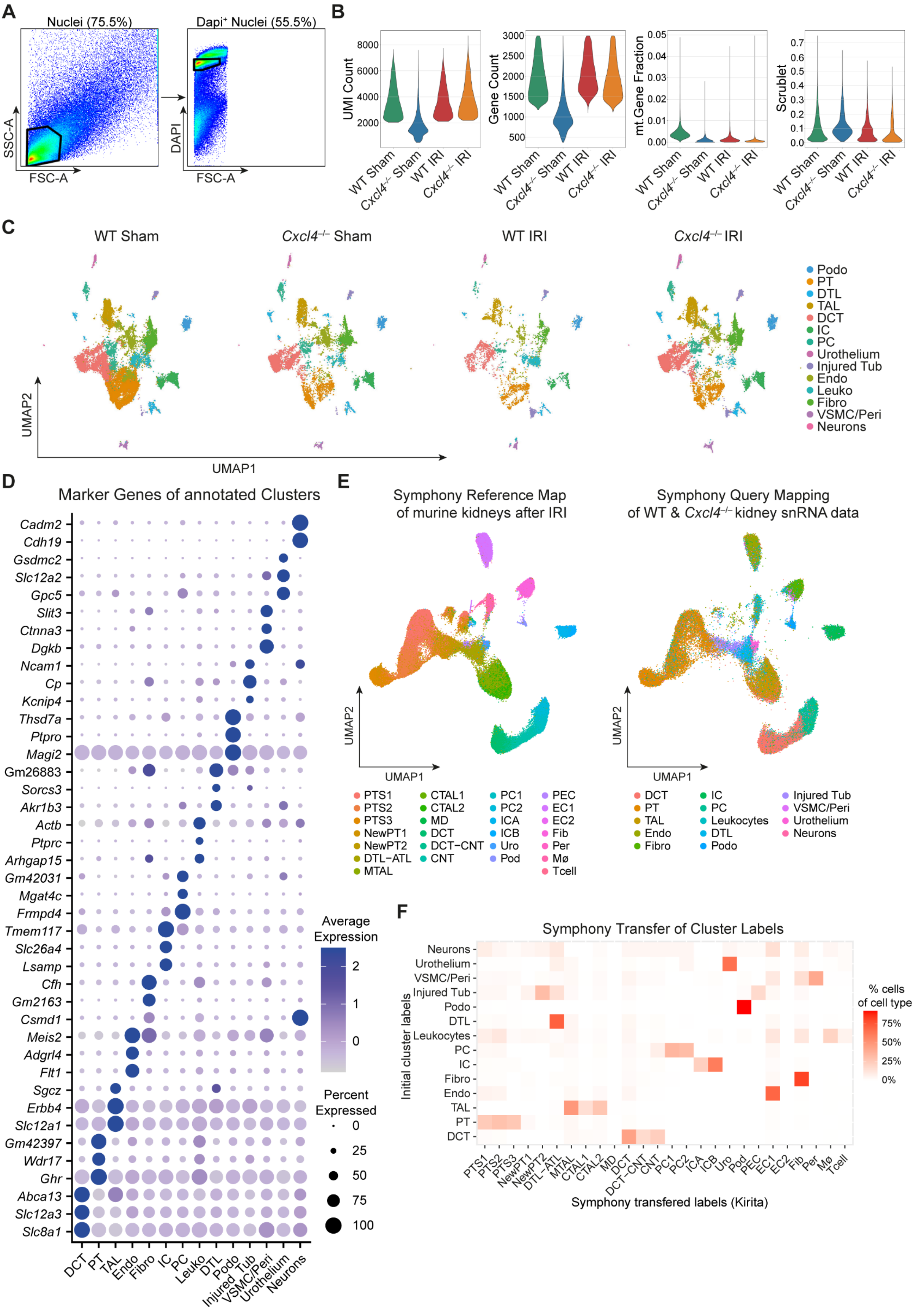
A
Nuclei (75.5%)
Dapi⁺ Nuclei (55.5%)
SSC-A
FSC-A
DAPI
FSC-A
B
UMI Count
Gene Count
mt.Gene Fraction
Scrublet
WT Sham
Cxcl4⁻/⁻ Sham
WT IRI
Cxcl4⁻/⁻ IRI
C
WT Sham
Cxcl4⁻/⁻ Sham
WT IRI
Cxcl4⁻/⁻ IRI
Podo
PT
DTL
TAL
DCT
IC
PC
Urothelium
Injured Tub
Endo
Leuko
Fibro
VSMC/Peri
Neurons
UMAP2
UMAP1
D
Marker Genes of annotated Clusters
Cadm2
Cdh19
Gsdmc2
Slc12a2
Gpc5
Slit3
Ctnna3
Dgkb
Ncam1
Cp
Kcnip4
Thsd7a
Ptpro
Magi2
Gm26883
Sorcs3
Akr1b3
Actb
Ptprc
Arhgap15
Gm42031
Mgat4c
Frmpd4
Tmem117
Slc26a4
Lsamp
Cfh
Gm2163
Csmd1
Meis2
Adgrl4
Flt1
Sgcz
Erbb4
Slc12a1
Gm42397
Wdr17
Ghr
Abca13
Slc12a3
Slc8a1
DCT
PT
TAL
Endo
Fibro
IC
PC
Leuko
DTL
Podo
Injured Tub
VSMC/Peri
Urothelium
Neurons
Average Expression
Percent Expressed
E
Symphony Reference Map of murine kidneys after IRI
Symphony Query Mapping of WT & Cxcl4⁻/⁻ kidney snRNA data
UMAP2
UMAP1
PTS1
PTS2
PTS3
NewPT1
NewPT2
DTL−ATL
MTAL
CTAL1
CTAL2
MD
DCT
DCT−CNT
CNT
PC1
PC2
ICA
ICB
Uro
Pod
PEC
EC1
EC2
Fib
Per
Mø
Tcell
DCT
PT
TAL
Endo
Fibro
IC
PC
Leukocytes
DTL
Podo
Injured Tub
VSMC/Peri
Urothelium
Neurons
F
Symphony Transfer of Cluster Labels
Initial cluster labels
Neurons
Urothelium
VSMC/Peri
Injured Tub
Podo
DTL
Leukocytes
PC
IC
Fibro
Endo
TAL
PT
DCT
% cells of cell type
75%
50%
25%
0%
PTS1
PTS2
PTS3
NewPT1
NewPT2
DTL−ATL
MTAL
CTAL1
CTAL2
MD
DCT
DCT−CNT
CNT
PC1
PC2
ICA
ICB
Uro
Pod
PEC
EC1
EC2
Fib
Per
Mø
Tcell
Symphony transfered labels (Kirita)

Supplemental Figure 3: Quality Control of single nuclear RNA sequencing of WT and *Cxcl4*$^{-/-}$ mice after IRI or sham surgery. Related to Fig. 3. A) Representative flow cytometric plots for isolation of DAPI^{+} nuclei. **B)** UMI Count, gene count, mitochondrial gene (mt.gene) fraction as well as scrublet doublet score for individual snRNA-seq libraries. **C)** UMAP embedding of 66235 nuclei split by genotype and surgery. Labels refer to clusters. Podo: podocytes, PT: proximal tubule, DTL: descending thin limb, TAL: thick ascending limb, DCT: distal convoluted tubule, IC: intercalated cells, PC: principal cells, DME: deep medullary epithelium, Injured Tub: injured tubular cells, Endo: endothelial cells, Fibro: fibroblasts, VSMC: vascular smooth muscle cells, Peri: pericytes. **D)** Dotplot of the top 3 specific genes for cell clusters shown in Fig. 3A. **E)** Symphony Reference Map of murine kidneys after IRI from Kirita et al. with Query Mapping of kidneys from WT or *Cxcl4*$^{-/-}$ mice after sham or IRI surgery into the same low dimensional space. **F)** Symphony Transfer of Cluster Labels from Kirita et al.

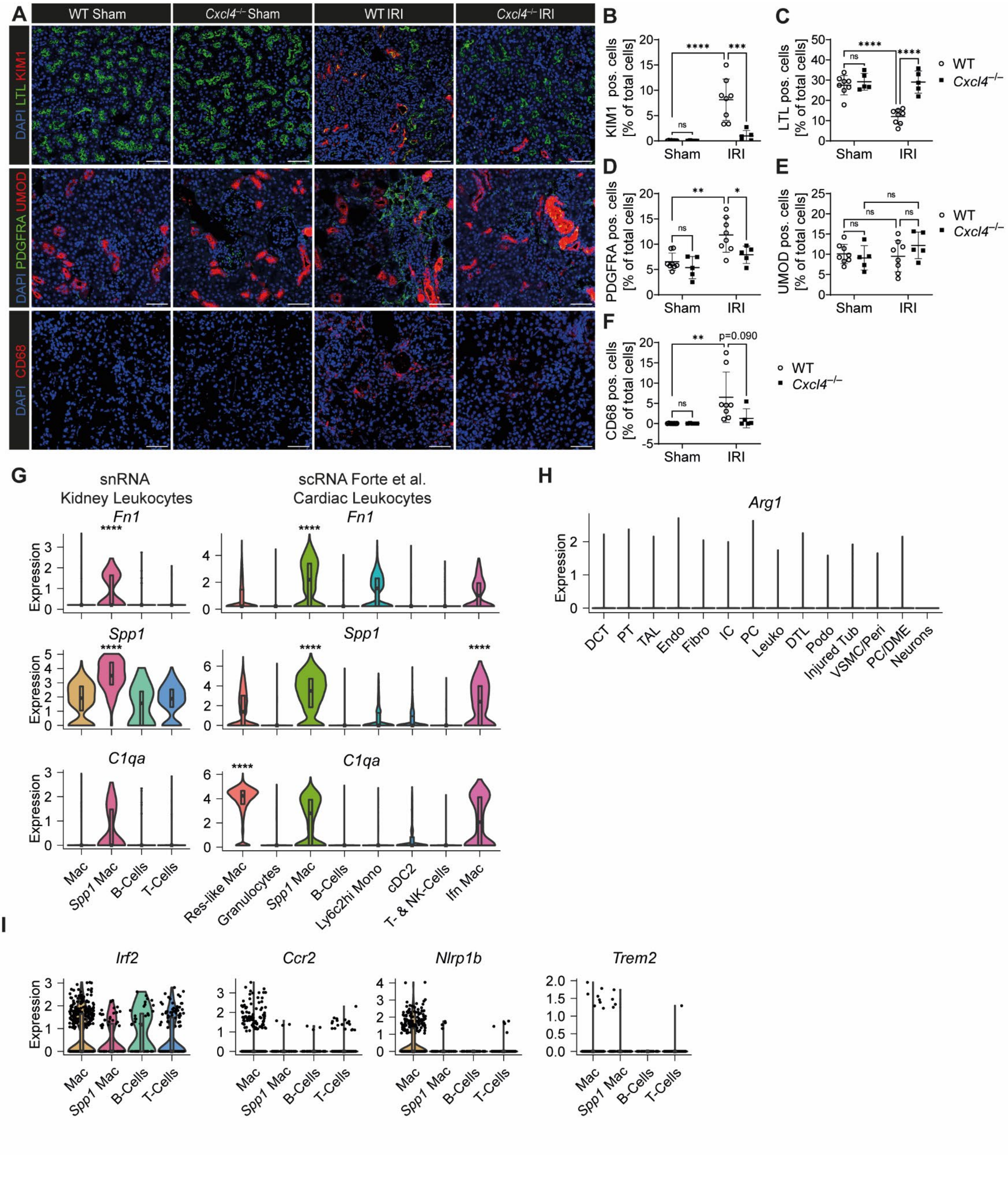

A
WT Sham
Cxcl4−/− Sham
WT IRI
Cxcl4−/− IRI
DAPI LTL KIM1
DAPI PDGFRA UMOD
DAPI CD68
B
KIM1 pos. cells [% of total cells]
C
LTL pos. cells [% of total cells]
D
PDGFRA pos. cells [% of total cells]
E
UMOD pos. cells [% of total cells]
F
CD68 pos. cells [% of total cells]
p=0.090
Sham
IRI
WT
Cxcl4−/−
G
snRNA Kidney Leukocytes
scRNA Forte et al. Cardiac Leukocytes
Fn1
Spp1
C1qa
Expression
Mac
Spp1 Mac
B-Cells
T-Cells
Res-like Mac
Granulocytes
Ly6c2hi Mono
cDC2
T- & NK-Cells
Ifn Mac
H
Arg1
DCT
PT
TAL
Endo
Fibro
IC
PC
Leuko
DTL
Podo
Injured Tub
VSMC/Peri
PC/DME
Neurons
I
Irf2
Ccr2
Nlrp1b
Trem2

Supplemental Figure 4: Compositional validation and marker gene analysis of single nuclear RNA sequencing data of WT and *Cxcl4*$^{-/-}$ mice after IRI or sham surgery. Related to Fig. 3. A) Representative images of immunofluorescence-stained sections for Lotus tetragonolobus lectin (LTL) and Kidney Injury Molecule -1 (KIM1), PDGFRA and Uromodulin (UMOD) or CD68 from WT or *Cxcl4*$^{-/-}$ kidneys after Sham or IRI surgery. Scale bar = 50µm. **B)** Quantification of KIM1^{+} injured tubular cells based on the staining in A. **C)** Quantification of LTL^{+} proximal tubular cells based on the staining in A. **D)** Quantification of PDGFRA^{+} fibroblasts based on the staining in A. **E)** Quantification of Uromodulin^{+} thick ascending loop cells based on the staining in A. **F)** Quantification of CD68^{+} macrophages based on the staining in A. **G)** *Fn1*, *Spp1*, and *C1qa* expression stratified by immune cell cluster for sub-clustered renal snRNA leukocytes as well as sub-clustered cardiac immune cells from Forte et al. **H)** snRNA-seq *Arg1* expression stratified by clusters. **I)** *Irf2*, *Ccr2*, *Nlrp1b*, and *Trem2* expression in renal snRNA leukocytes stratified by immune cell clusters. For B-F a two-way ANOVA was computed using Tukey corrections. For G p-values from MAST (Seurat, FindAllMarkers) are displayed. *P<0.05, **P<0.01, ***P<0.001, ****P<0.0001. For B-F n = 8 WT Sham ; 5 *Cxcl4*$^{-/-}$ Sham; 8 WT IRI; 5 *Cxcl4*$^{-/-}$ IRI.

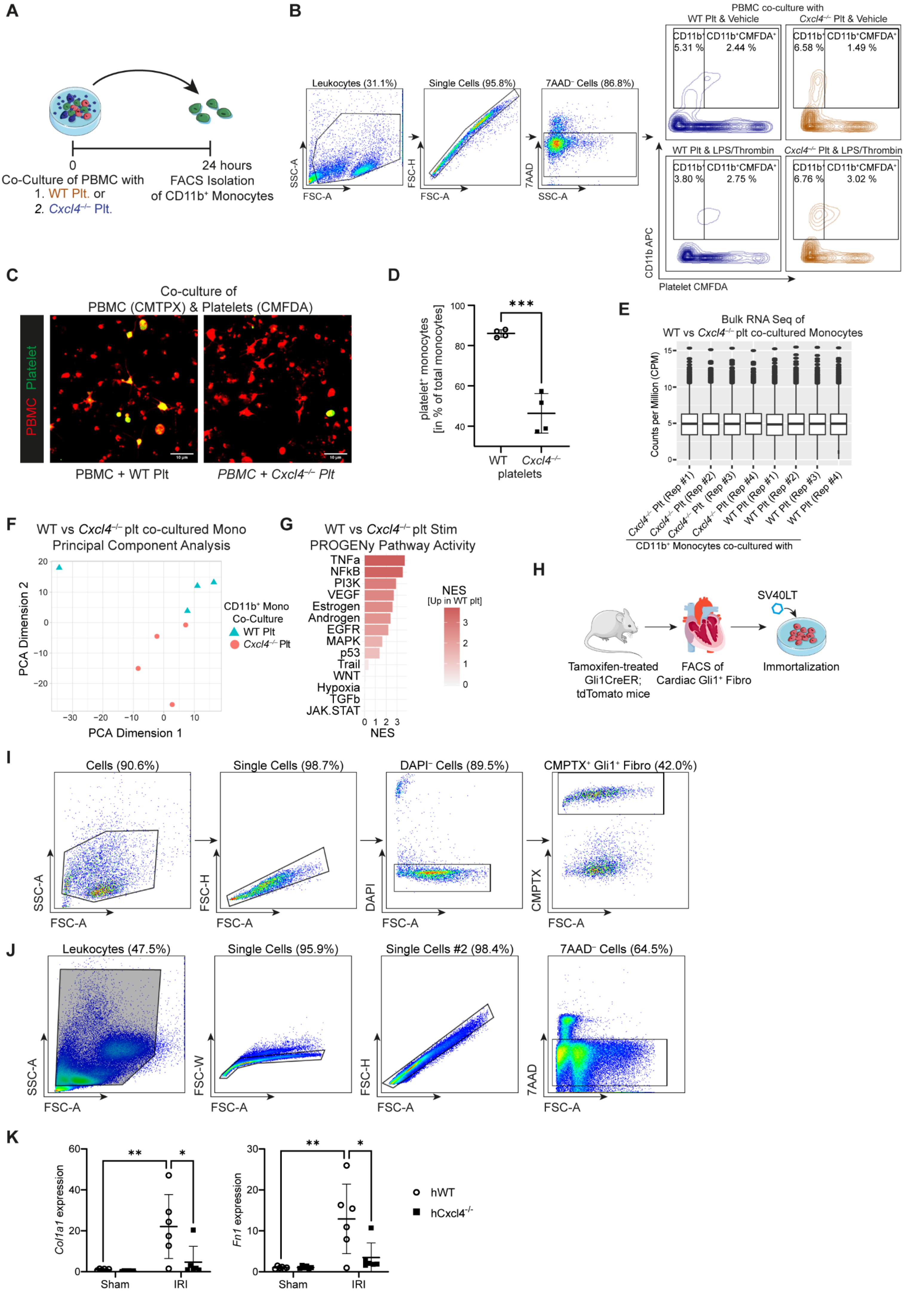
A
B
C
D
E
F
G
H
I
J
K
0
24 hours
Co-Culture of PBMC with
1. WT Plt. or
2. Cxcl4−/− Plt.
FACS Isolation
of CD11b+ Monocytes
Leukocytes (31.1%)
Single Cells (95.8%)
7AAD− Cells (86.8%)
PBMC co-culture with
WT Plt & Vehicle
Cxcl4−/− Plt & Vehicle
WT Plt & LPS/Thrombin
Cxcl4−/− Plt & LPS/Thrombin
CD11b+ 5.31 %
CD11b+CMFDA+ 2.44 %
CD11b+ 6.58 %
CD11b+CMFDA+ 1.49 %
CD11b+ 3.80 %
CD11b+CMFDA+ 2.75 %
CD11b+ 6.76 %
CD11b+CMFDA+ 3.02 %
CD11b APC
Platelet CMFDA
Co-culture of
PBMC (CMTPX) & Platelets (CMFDA)
PBMC Platelet
PBMC + WT Plt
PBMC + Cxcl4−/− Plt
platelet+ monocytes [in % of total monocytes]
WT
Cxcl4−/−
platelets
Bulk RNA Seq of
WT vs Cxcl4−/− plt co-cultured Monocytes
Counts per Million (CPM)
Cxcl4−/− Plt (Rep #1)
Cxcl4−/− Plt (Rep #2)
Cxcl4−/− Plt (Rep #3)
Cxcl4−/− Plt (Rep #4)
WT Plt (Rep #1)
WT Plt (Rep #2)
WT Plt (Rep #3)
WT Plt (Rep #4)
CD11b+ Monocytes co-cultured with
WT vs Cxcl4−/− plt co-cultured Mono
Principal Component Analysis
PCA Dimension 1
PCA Dimension 2
CD11b+ Mono
Co-Culture
WT Plt
Cxcl4−/− Plt
WT vs Cxcl4−/− plt Stim
PROGENy Pathway Activity
TNFa
NFkB
PI3K
VEGF
Estrogen
Androgen
EGFR
MAPK
p53
Trail
WNT
Hypoxia
TGFb
JAK.STAT
NES
NES
[Up in WT plt]
SV40LT
Tamoxifen-treated
Gli1CreER;
tdTomato mice
FACS of
Cardiac Gli1+ Fibro
Immortalization
Cells (90.6%)
Single Cells (98.7%)
DAPI− Cells (89.5%)
CMPTX+ Gli1+ Fibro (42.0%)
Leukocytes (47.5%)
Single Cells (95.9%)
Single Cells #2 (98.4%)
7AAD− Cells (64.5%)
Col1a1 expression
Fn1 expression
Sham
IRI
hWT
hCxcl4−/−

Supplemental Figure 5: *Cxcl4* is critical for platelet-monocyte interaction and mediates profibrotic fibroblast activation. Related to Figs. 4 and 5. A) Experimental design of murine PBMC co-culture with WT or *Cxcl4*$^{-/-}$ platelets with subsequent FACS isolation of CD11b$^+$ monocytes for Bulk RNA sequencing. **B)** Representative flow cytometric plots for quantification of APC$^+$CD11b$^+$ monocytes in WT PBMC co-cultured with CMFDA$^+$ WT or *Cxcl4*$^{-/-}$ platelets (Plt) after stimulation with Vehicle or LPS and Thrombin. **C)** Confocal imaging of CMPTX$^+$ WT PBMC after 48 hours co-culture with CMFDA$^+$ WT or *Cxcl4*$^{-/-}$ platelets. Scale Bar=10 µm. **D)** Automated quantification of CMFDA$^+$CMPTX$^+$ plate-adherent platelet-monocytes aggregates compared to total plate-adherent monocytes from (B) [n = 4]. **E)** Counts per Million (CPM) for individual samples of FACS-isolated CD11b$^+$ monocytes co-cultured with either WT or *Cxcl4*$^{-/-}$ platelets. **F)** Generalised (glmpca) Principal Component Analysis (PCA) of FACS-isolated CD11b$^+$ monocytes co-cultured with either WT or *Cxcl4*$^{-/-}$ platelets. **G)** PROGENy analysis of differentially expressed genes in CD11b$^+$ monocytes co-cultured with either WT or *Cxcl4*$^{-/-}$ platelets. **H)** Schematic for isolation and immortalization of cardiac Gli1$^+$ fibroblasts. **I)** Representative flow cytometric plots for FACS of CMPTX$^+$Gli1$^+$ fibroblasts (CMPTX$^+$Gli1$^+$ Fibro). **J)** Gating strategy and representative flow cytometric plots for analysis of CXCL4 expression in peripheral blood as shown in Fig 5B. **K)** RT-qPCR analysis for *Col1a1* and *Fn1* in IRI and sham kidneys of ^{HSC}WT and ^{HSC}*Cxcl4*$^{-/-}$ mice. All quantitative data are shown as mean±SD. For D a two-tailed unpaired t-test was performed. For K a two-way ANOVA was computed using Tukey correction for multiple testing. *P<0.05, **P<0.01, ***P<0.001.

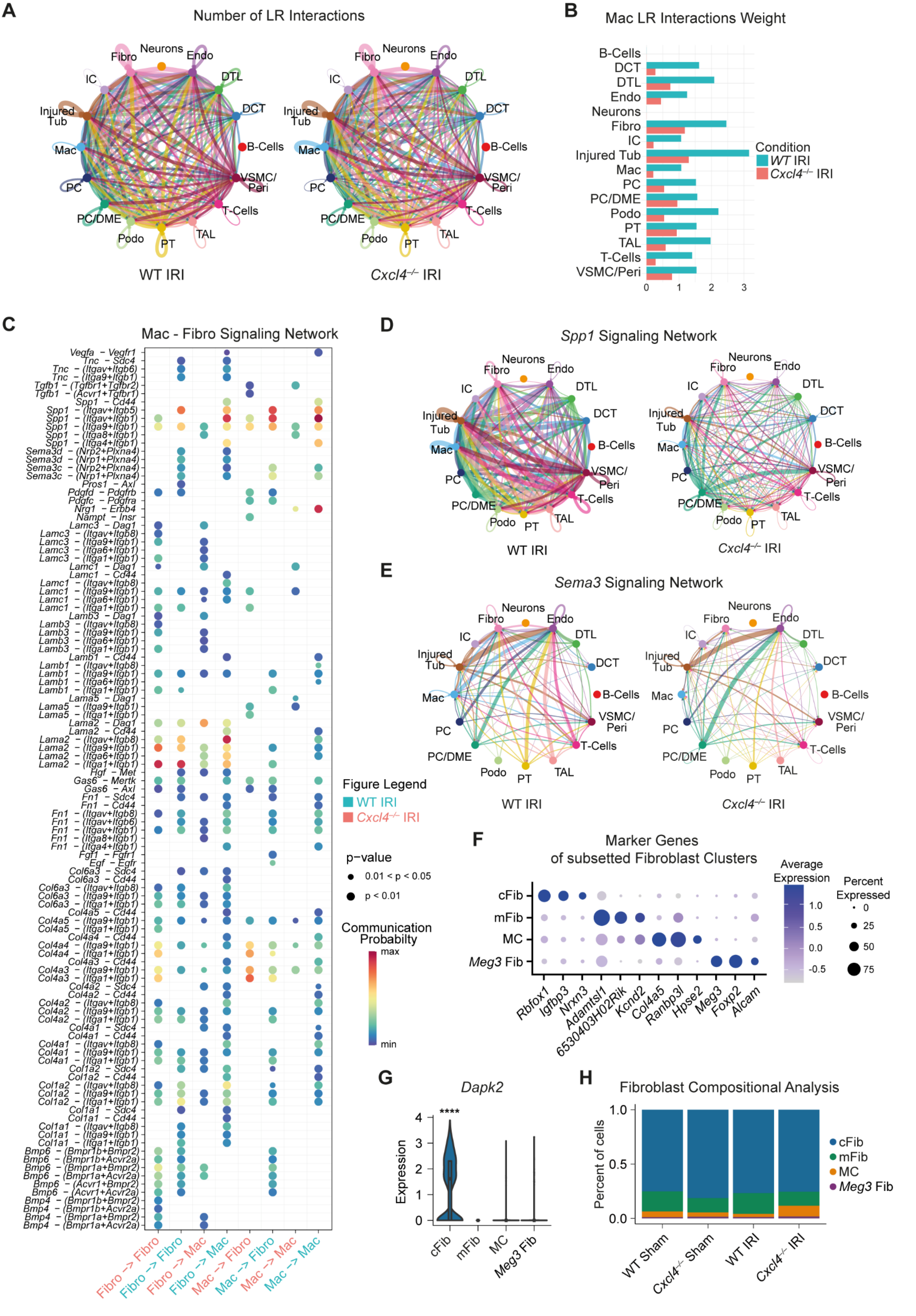
A
Number of LR Interactions
Neurons
Fibro
Endo
IC
DTL
Injured Tub
DCT
B-Cells
Mac
VSMC/ Peri
PC
T-Cells
PC/DME
TAL
Podo
PT
WT IRI
Cxcl4−/− IRI
B
Mac LR Interactions Weight
B-Cells
DCT
DTL
Endo
Neurons
Fibro
IC
Injured Tub
Mac
PC
PC/DME
Podo
PT
TAL
T-Cells
VSMC/Peri
0
1
2
3
Condition
WT IRI
Cxcl4−/− IRI
C
Mac - Fibro Signaling Network
Vegfa − Vegfr1
Tnc − Sdc4
Tnc − (Itgav+Itgb6)
Tnc − (Itga9+Itgb1)
Tgfb1 − (Tgfbr1+Tgfbr2)
Tgfb1 − (Acvr1+Tgfbr1)
Spp1 − Cd44
Spp1 − (Itgav+Itgb5)
Spp1 − (Itgav+Itgb1)
Spp1 − (Itga9+Itgb1)
Spp1 − (Itga8+Itgb1)
Spp1 − (Itga4+Itgb1)
Sema3d − (Nrp2+Plxna4)
Sema3d − (Nrp1+Plxna4)
Sema3c − (Nrp2+Plxna4)
Sema3c − (Nrp1+Plxna4)
Pros1 − Axl
Pdgfd − Pdgfrb
Pdgfc − Pdgfra
Nrg1 − Erbb4
Nampt − Insr
Lamc3 − Dag1
Lamc3 − (Itgav+Itgb8)
Lamc3 − (Itga9+Itgb1)
Lamc3 − (Itga6+Itgb1)
Lamc3 − (Itga1+Itgb1)
Lamc1 − Dag1
Lamc1 − Cd44
Lamc1 − (Itgav+Itgb8)
Lamc1 − (Itga9+Itgb1)
Lamc1 − (Itga6+Itgb1)
Lamc1 − (Itga1+Itgb1)
Lamb3 − Dag1
Lamb3 − (Itgav+Itgb8)
Lamb3 − (Itga9+Itgb1)
Lamb3 − (Itga6+Itgb1)
Lamb3 − (Itga1+Itgb1)
Lamb1 − Cd44
Lamb1 − (Itgav+Itgb8)
Lamb1 − (Itga9+Itgb1)
Lamb1 − (Itga6+Itgb1)
Lamb1 − (Itga1+Itgb1)
Lama5 − Dag1
Lama5 − (Itga9+Itgb1)
Lama5 − (Itga1+Itgb1)
Lama2 − Dag1
Lama2 − Cd44
Lama2 − (Itgav+Itgb8)
Lama2 − (Itga9+Itgb1)
Lama2 − (Itga6+Itgb1)
Lama2 − (Itga1+Itgb1)
Hgf − Met
Gas6 − Mertk
Gas6 − Axl
Fn1 − Sdc4
Fn1 − Cd44
Fn1 − (Itgav+Itgb8)
Fn1 − (Itgav+Itgb6)
Fn1 − (Itgav+Itgb1)
Fn1 − (Itga8+Itgb1)
Fn1 − (Itga4+Itgb1)
Fgf1 − Fgfr1
Egf − Egfr
Col6a3 − Sdc4
Col6a3 − Cd44
Col6a3 − (Itgav+Itgb8)
Col6a3 − (Itga9+Itgb1)
Col6a3 − (Itga1+Itgb1)
Col4a5 − Cd44
Col4a5 − (Itga9+Itgb1)
Col4a5 − (Itga1+Itgb1)
Col4a4 − Cd44
Col4a4 − (Itga9+Itgb1)
Col4a4 − (Itga1+Itgb1)
Col4a3 − Cd44
Col4a3 − (Itga9+Itgb1)
Col4a3 − (Itga1+Itgb1)
Col4a2 − Sdc4
Col4a2 − Cd44
Col4a2 − (Itgav+Itgb8)
Col4a2 − (Itga9+Itgb1)
Col4a2 − (Itga1+Itgb1)
Col4a1 − Sdc4
Col4a1 − Cd44
Col4a1 − (Itgav+Itgb8)
Col4a1 − (Itga9+Itgb1)
Col4a1 − (Itga1+Itgb1)
Col1a2 − Sdc4
Col1a2 − Cd44
Col1a2 − (Itgav+Itgb8)
Col1a2 − (Itga9+Itgb1)
Col1a2 − (Itga1+Itgb1)
Col1a1 − Sdc4
Col1a1 − Cd44
Col1a1 − (Itgav+Itgb8)
Col1a1 − (Itga9+Itgb1)
Col1a1 − (Itga1+Itgb1)
Bmp6 − (Bmpr1b+Bmpr2)
Bmp6 − (Bmpr1b+Acvr2a)
Bmp6 − (Bmpr1a+Bmpr2)
Bmp6 − (Bmpr1a+Acvr2a)
Bmp6 − (Acvr1+Bmpr2)
Bmp6 − (Acvr1+Acvr2a)
Bmp4 − (Bmpr1b+Bmpr2)
Bmp4 − (Bmpr1b+Acvr2a)
Bmp4 − (Bmpr1a+Bmpr2)
Bmp4 − (Bmpr1a+Acvr2a)
Fibro -> Fibro
Fibro -> Fibro
Fibro -> Mac
Fibro -> Mac
Mac -> Fibro
Mac -> Fibro
Mac -> Mac
Mac -> Mac
Figure Legend
WT IRI
Cxcl4−/− IRI
p−value
0.01 < p < 0.05
p < 0.01
Communication Probabilty
max
min
D
Spp1 Signaling Network
WT IRI
Cxcl4−/− IRI
E
Sema3 Signaling Network
WT IRI
Cxcl4−/− IRI
F
Marker Genes of subsetted Fibroblast Clusters
cFib
mFib
MC
Meg3 Fib
Rbfox1
Igfbp3
Nrxn3
Adamtsl1
6530403H02Rik
Kcnd2
Col4a5
Ranbp3l
Hpse2
Meg3
Foxp2
Alcam
Average Expression
1.0
0.5
0.0
-0.5
Percent Expressed
0
25
50
75
G
Dapk2
****
Expression
0
1
2
3
4
cFib
mFib
MC
Meg3 Fib
H
Fibroblast Compositional Analysis
Percent of cells
0.0
0.5
1.0
WT Sham
Cxcl4−/− Sham
WT IRI
Cxcl4−/− IRI
cFib
mFib
MC
Meg3 Fib

Supplemental Figure 6: Ligand Receptor Interaction analysis of snRNA WT and *Cxcl4*$^{-/-}$ kidneys after IRI. Related to Fig. 6. A) Network plots of inferred number of ligand receptor interactions split by genotype. Labels refer to clusters. DCT: distal convoluted tubule, Endo: endothelial cells, Fibro: fibroblasts, IC: intercalated cells, Tub: tubular cells, Mac: macrophages, PC: principal cells, Podo: podocytes, PT: proximal tubular cells, TAL: thick ascending limb, VSMC: vascular smooth muscle cells, Peri: pericytes. **B)** Inferred macrophage (Mac) Ligand-Receptor (LR) interaction weight stratified by cell clusters and genotype. Labels refer to clusters. **C)** Dot-Plot for all inferred ligand receptor interactions of macrophages and fibroblasts stratified by genotype and cell-cell interaction. **D)** Network plots for inferred *Spp1* ligand receptor interaction activity split by genotype. **E)** Network plots for inferred *Sema3* ligand receptor interaction activity split by genotype. **F)** Dotplot of the top 3 specific genes for fibroblast sub-clusters shown in Fig. 5F. **G**) *Dapk2* Expression stratified by fibroblast sub-clusters. **H)** Fibroblast sub-cluster composition in percent stratified by genotype and surgery. For G p-values from MAST (Seurat, FindAllMarkers) are displayed. ****P<0.0001.

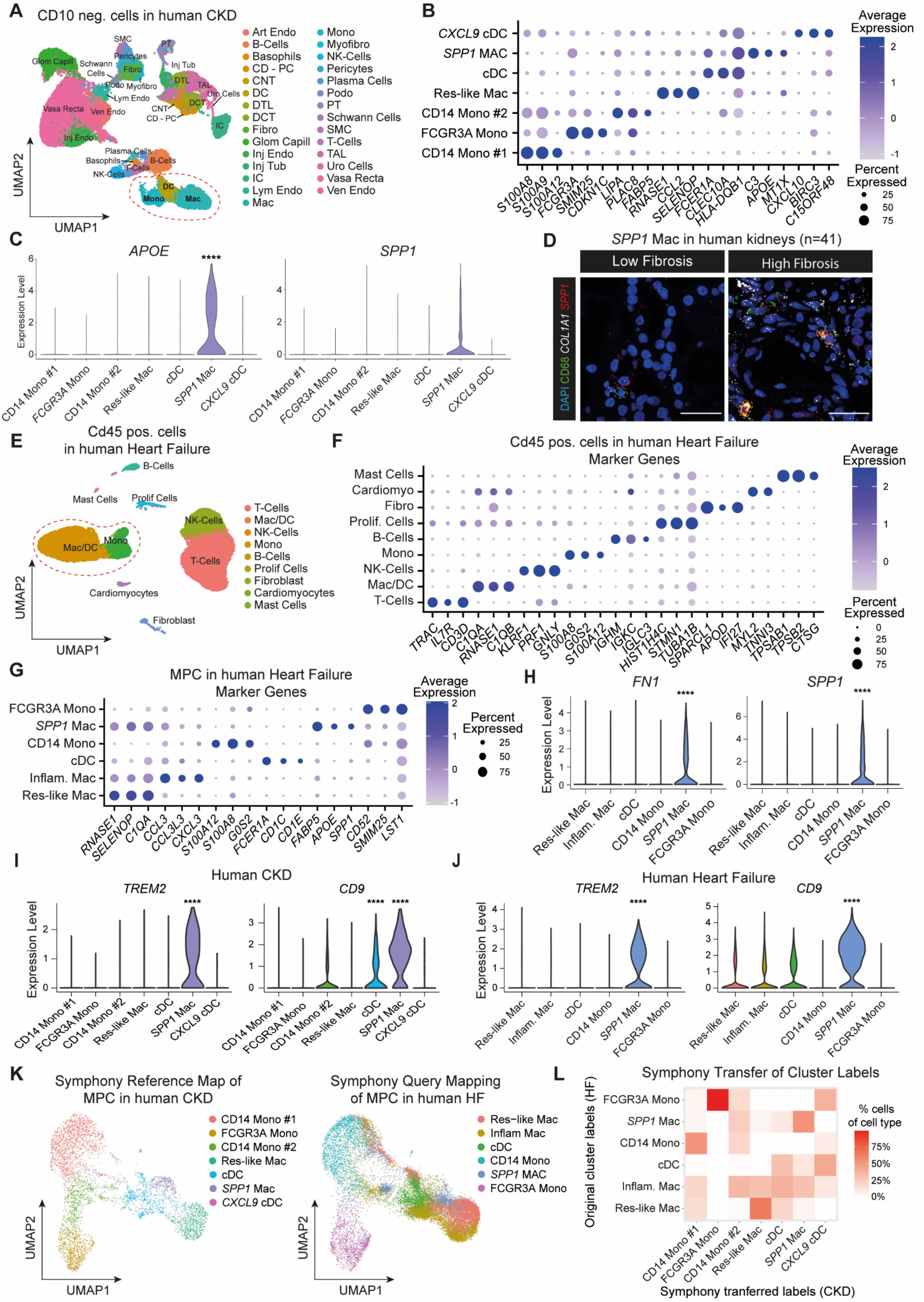
A
CD10 neg. cells in human CKD
Art Endo
B-Cells
Basophils
CD - PC
CNT
DC
DTL
DCT
Fibro
Glom Capill
Inj Endo
Inj Tub
IC
Lym Endo
Mac
Mono
Myofibro
NK-Cells
Pericytes
Plasma Cells
Podo
PT
Schwann Cells
SMC
T-Cells
TAL
Uro Cells
Vasa Recta
Ven Endo
UMAP1
UMAP2
B
CXCL9 cDC
SPP1 MAC
cDC
Res-like Mac
CD14 Mono #2
FCGR3A Mono
CD14 Mono #1
S100A8
S100A9
S100A12
FCGR3A
SMIM25
CDKN1C
LIPA
PLAC8
FABP5
RNASE1
CCL2
SELENOP
FCER1A
CLEC10A
HLA-DQB1
C3
APOE
MT1X
CXCL10
BIRC3
C15ORF48
Average Expression
Percent Expressed
C
APOE
SPP1
Expression Level
****
CD14 Mono #1
FCGR3A Mono
CD14 Mono #2
Res-like Mac
cDC
SPP1 Mac
CXCL9 cDC
D
SPP1 Mac in human kidneys (n=41)
Low Fibrosis
High Fibrosis
DAPI CD68 COL1A1 SPP1
E
Cd45 pos. cells
in human Heart Failure
T-Cells
Mac/DC
NK-Cells
Mono
B-Cells
Prolif Cells
Fibroblast
Cardiomyocytes
Mast Cells
UMAP1
UMAP2
F
Cd45 pos. cells in human Heart Failure
Marker Genes
Mast Cells
Cardiomyo
Fibro
Prolif. Cells
B-Cells
Mono
NK-Cells
Mac/DC
T-Cells
TRAC
IL7R
CD3D
C1QA
RNASE1
C1QB
KLRF1
PRF1
GNLY
S100A8
G0S2
S100A12
IGHM
IGKC
IGLC3
HIST1H4C
STMN1
TUBA1B
SPARCL1
APOD
IFI27
MYL2
TNNI3
TPSAB1
TPSB2
CTSG
Average Expression
Percent Expressed
G
MPC in human Heart Failure
Marker Genes
FCGR3A Mono
SPP1 Mac
CD14 Mono
cDC
Inflam. Mac
Res-like Mac
RNASE1
SELENOP
C1QA
CCL3
CCL3L3
CXCL3
S100A12
S100A8
G0S2
FCER1A
CD1C
CD1E
FABP5
APOE
SPP1
CD52
SMIM25
LST1
Average Expression
Percent Expressed
H
FN1
SPP1
Expression Level
****
Res-like Mac
Inflam. Mac
cDC
CD14 Mono
SPP1 Mac
FCGR3A Mono
I
Human CKD
TREM2
CD9
Expression Level
****
J
Human Heart Failure
TREM2
CD9
Expression Level
****
K
Symphony Reference Map of
MPC in human CKD
CD14 Mono #1
FCGR3A Mono
CD14 Mono #2
Res-like Mac
cDC
SPP1 Mac
CXCL9 cDC
Symphony Query Mapping
of MPC in human HF
Res−like Mac
Inflam Mac
cDC
CD14 Mono
SPP1 MAC
FCGR3A Mono
UMAP1
UMAP2
L
Symphony Transfer of Cluster Labels
Original cluster labels (HF)
FCGR3A Mono
SPP1 Mac
CD14 Mono
cDC
Inflam. Mac
Res-like Mac
% cells of cell type
75%
50%
25%
0%
CD14 Mono #1
FCGR3A Mono
CD14 Mono #2
Res-like Mac
cDC
SPP1 Mac
CXCL9 cDC
Symphony tranferred labels (CKD)

Supplemental Figure 7: Single cell RNA analysis of MPC in human CKD and heart failure. Related to Fig. 7. A) UMAP of all (51849) CD10 negative cells from Kuppe et al.. Mononuclear phagocytes (MPC, circled in red) were sub-clustered for further analysis. Labels refer to clusters. Art Endo: arterial endothelium. CD - PC: Collecting Duct - Principal cells, CNT: connecting tubule, DC: dendritic cells, DTL: descending thin limb, DCT: distal convoluted tubule, Fibro: fibroblast, Glom Capill: glomerular capillaries, Inj Endo: injured endothelium, Inj Tub: injured tubule, IC: intercalated cells, Lymp Endo: lymphatic endothelium, Mac: macrophages, Mono: monocytes, Myofibro: myofibroblasts, Podo: podocytes, PT: proximal tubule, SMC: smooth muscle cells, TAL: thick ascending limb, Uro Cells: urothelial cells, Ven Endo: venular endothelium. **B)** Dotplot of the top 3 specific genes for MPC cell clusters shown in Fig. 6A. Mono: monocytes, Mac: macrophages, cDC: conventional dendritic cells, Res-like Mac: Resident-like macrophages. **C)** *APOE* and *SPP1* expression in kidney MPC stratified by cell type. **D)** RNA-ISH for *SPP1* and *COL1A1* with CD68 immunofluorescent staining in human kidney nephrectomies (n=41). Representative images of kidneys with low and high fibrosis as determined by *COL1A1* expression are shown. *SPP1*$^{+}$CD68^{+} macrophages are circled in white, while *SPP1*$^{-}$CD68^{+} macrophages are circled in orange. Scale bar = 30 µm. **E)** UMAP of all (58786) immune cells from Rao et al.. MPC (circled in red) were sub-clustered for further analysis. Labels refer to clusters. Mac: macrophages, DC: dendritic cells, Mono: monocytes, Prolif Cells: proliferating cells. **F)** Dotplot of the top 3 specific genes for immune cell clusters shown in E. **G)** Dotplot of the top 3 specific genes for MPC clusters shown in Fig. 6E. **H)** *FN1* and *SPP1* expression in cardiac MPC stratified by cell type. **(I)** *TREM2* and CD9 expression in human kidney MPC stratified by cell type. **(J)** *TREM2* and *CD9* expression in human cardiac MPC stratified by cell type. **K)** Symphony Reference Map of renal immune cells in human chronic kidney disease (CKD) with Query Mapping of cardiac immune cells in human heart failure (HF) from Rao et al. into the same low dimensional space. **L)** Symphony Transfer of Cluster Labels. For C, H, I and J p-values from MAST (Seurat, FindAllMarkers) are displayed. ****P<0.0001.
